# Effects of Geological and Environmental Events on the Diversity and Genetic Divergence of Four Closely Related Pines: *Pinus koraiensis*, *P. armandii*, *P. griffithii*, and *P. pumila*

**DOI:** 10.3389/fpls.2018.01264

**Published:** 2018-08-28

**Authors:** Yun Jia, Juan Zhu, Ying Wu, Wei-Bing Fan, Gui-Fang Zhao, Zhong-Hu Li

**Affiliations:** Key Laboratory of Resource Biology and Biotechnology in Western China, Ministry of Education, College of Life Sciences, Northwest University, Xi’an, China

**Keywords:** genetic divergence, nucleotide polymorphism, *Pinus armandii*, *Pinus griffithii*, *Pinus koraiensis*, *Pinus pumila*

## Abstract

The effects of mountain uplift and environmental oscillations on nucleotide variability and species divergence remain largely unknown in East Asia. In this study, based on multiple nuclear DNA markers, we investigated the levels and patterns of nucleotide diversity and interspecific divergence in four closely related pines in China, i.e., *Pinus koraiensis*, *P. armandii*, *P. griffithii*, and *P. pumila*. The four pine taxa shared low levels of nucleotide polymorphisms at the species level. *P. pumila* had the highest silent nucleotide diversity (π_sil_ = 0.00661) whereas *P. griffithii* had the lowest (π_sil_ = 0.00175), while the levels of genetic polymorphism in *P. armandii* (π_sil_ = 0.00508) and *P. koraiensis* (π_sil_ = 0.00652) were intermediate between the other two species. Population genetic structure analysis showed that variations primarily existed within populations of the four pine species, presumably due to habitat fragmentation or the island-like distributions of *Pinus* species. Population divergence (*F*_ST_) analysis showed that the genetic divergence between *P. griffithii* and *P. koraiensis* was much greater than that between *P. koraiensis* and the other two pines species. Isolation-with-migration analysis suggested that asymmetric gene flow had occurred between any two pairs of pine species. Phylogenetic analyses indicated that the four allied species split into two groups about 1.37 million years ago, where *P*. *armandii* and *P*. *pumila* were closer and clustered as sister species, whereas *P*. *koraiensis* and *P*. *griffithii* were clustered on another branch. Our results and those obtained in previous studies suggest that mountain uplift and geological climate oscillations may have led to the patterns of genetic divergence and nucleotide variations in these four pine species.

## Introduction

Nucleotide diversity levels within populations and spatial patterns, as well as species divergence are of great importance in the field of evolutionary biology (Coyne and Orr, 2004; Hao et al., 2015; Ortego et al., 2015). Mountain uplift and past environmental oscillations may have been largely responsible for shaping the spatial patterns of diversity and genetic divergence among species (Coyne and Orr, 2004; Wachowiak et al., 2009). In general, the level and distribution of nucleotide diversity are historical products of the long-term evolution of a species, and they are largely associated with the evolutionary potential or future fate of a species (Wright and Gaut, 2005; Wachowiak et al., 2009; Zhou et al., 2014; Tsuda et al., 2017). In addition, ecological or proximal causes (e.g., mating systems) and various barriers (e.g., geographic and spatio-temporal isolation) due to geological history can cause fragmented of species distributions, which may lead to reduced gene flow between isolated populations and adaptability. This process initiates allopatric divergence, and local adaptation can ultimately drive populations toward speciation and change evolutionary processes (Cutter and Gray, 2016; Ren et al., 2017).

Conifers are anemophilous and outcrossing (Fu et al., 1999). They are mainly characterized by long life cycles, large effective population sizes, incomplete lineage sorting, and extensive introgression/hybridization among populations, which makes their genetic structure and spatial patterns of diversity very different from those found in traditional model plants (Neale and Kremer, 2011; Gao et al., 2012; Li et al., 2012, 2013; Hao et al., 2015). For instance, conifers tend to share haplotypes/genotypes among species, with no distinct genetic divergence across species ranges, and most of the genetic variations are found within populations (Willyard et al., 2009; Chen et al., 2010; Ren et al., 2012; Liu et al., 2014; Zhou et al., 2014). In recent years, many studies have determined nucleotide polymorphisms and speciation history patterns using multiple nuclear loci (Ma et al., 2006; Li et al., 2010; Gao et al., 2012; Wachowiak et al., 2013; Zhou et al., 2014; Zou et al., 2016). These biparentally inherited nuclear genes are functional genes that encode proteins and they are characterized by their orthology, moderate to high rates of evolution, and the presence of many phylogenetically informative sites (Zhou et al., 2014). Therefore, large numbers of nuclear markers can be used to detect the deep evolutionary relationships among closely related species, especially recently diverged taxa (Chen et al., 2010; Zou et al., 2016). In this study, we employed nucleotide polymorphisms as well as the population structure and speciation history to explore the relationships among four *Pinus* species.

Four related *Pinus* species in subsection *Strobus* occur in East Asia: *P. armandii* Franch., *P. koraiensis* Sieb. et Zucc., *P. griffithii* McClelland, and *P. pumila* (Pall.) Regel. These species share some common features, such as possessing five needle leaves in a bundle. There are obvious differences among these species in terms of their ecological niche, natural geographic distribution, morphology, wood anatomy, and cytology (Wu and Feng, 1995). *P. armandii*, *P. pumila*, and *P. koraiensis* occur according to the changes in the hydrothermal conditions, and *P. griffithii* is distributed on the China–Nepal and China–Bhutan borders (**Supplementary Figure S1**; Wu and Feng, 1995). The distributions of these pines also increase successively from low to high altitudes, where *P. koraiensis* occurs at altitudes of 150–1,800 m and *P. pumila* always forms copses with other coniferous trees on mountain tops at altitudes of 1,000–2,300 m. *P. armandii* usually occurs in pure forest or mixed forest at altitudes of 1,000–3,300 m, and *P. griffithii* is distributed in the same manner at altitudes of 1,600–3,300 m on the Qinghai–Tibet Plateau and Mount Everest. According to the classic categorization of *Pinus* sect. *Strobus*, *P. pumila* is categorized into the *P. koraiensis* taxon and *P. armandii* into the *P. griffithii* group (Fu et al., 1999). A study of the divergence of the resin ducts in *Pinus* sect. *Strobus* suggested that *P. armandii* is the most primitive species and its southward spread gave rise to *P. griffithii*, whereas its northward spread gave rise to *P. pumila* and *P. griffithii* (Peng, 1999). In recent years, several studies based on plastid molecular markers have shown that *P. koraiensis* and *P. pumila* are most closely related to each other (Peng, 1999; Wang et al., 2016). However, the accurate phylogenetic relationships and interspecific divergence among these related pine species is still controversial due to the limited availability of morphological and molecular biological evidence (Peng, 1999; Liu et al., 2014; Hao et al., 2015).

In addition, some studies found low variations in DNA barcodes, such as *rbc*L and *mat*K, among related species due to low levels of cpDNA diversity and genetic divergence (Syring et al., 2007; Li et al., 2015). Moreover, frequent interspecific introgression and hybridization among species have important effects on their genetic diversity levels, especially in parapatric or allopatric of species in China. Thus, in the current study, we used multiple nuclear genes to investigate the genetic diversity and divergence in four closely related pine species comprising *P. koraiensis*, *P. armandii*, *P. griffithii*, and *P. pumila*. We specifically addressed the following two questions. (1) How is the level and pattern of population divergence among the four pines species? (2) How is the pattern of gene flow and interspecific introgression between these closely related species in East Asia?

## Materials and Methods

### Population Sampling

**FIGURE 1:**
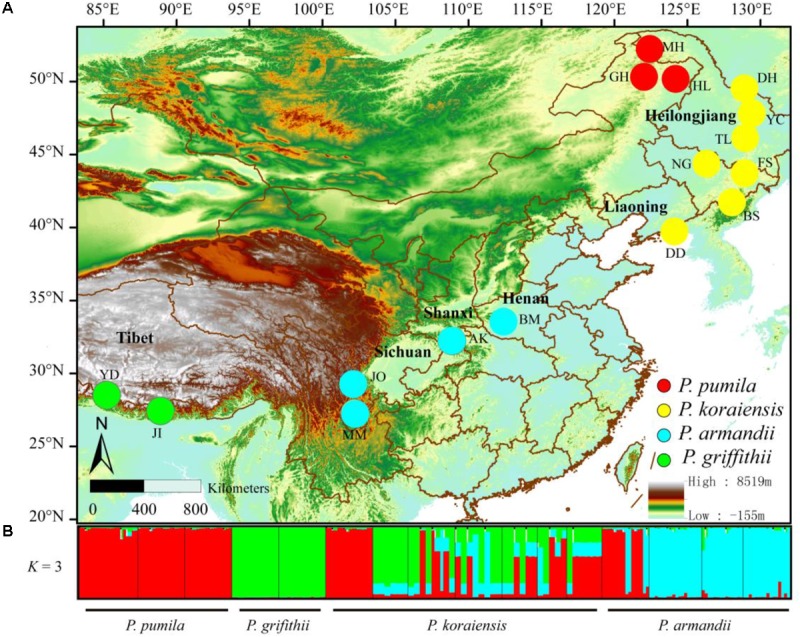
**(A)** Geographical distribution of the sampled populations of the four closely related species: *Pinus pumila* (*red*), *P. griffithii* (*green*), *P. koraiensis* (*yellow*), and *P. armandii* (*blue*). Color scales indicate different altitudes. **(B)** Bayesian clustering analysis to determine the population structure of the four pine species. *Red, green*, and *blue* represent the dominant clusters (*K* = 3) identified by STRUCTURE in each population.

To accurately determine the nucleotide diversity and interspecific relationships among pines, we sampled 216 individuals from 16 allopatric populations of the four pine species (**Figure 1**). The distance between any two trees of the same species was at least 50 m (**Supplementary Table S1**). We isolated the haploid megagametophyte from each of the sampled trees.

### DNA Extraction, PCR Amplification, and Sequencing

Total genomic DNA was extracted from the megagametophyte samples for each individual using the modified CTAB method (Doyle and Doyle, 1987). In preliminary studies, about 40 nuclear gene loci were screened for cross-amplification in the four pine species (Ma et al., 2006; Eckert et al., 2013). Finally, six polymorphic loci (1_1609_01, CL1694, PTIFG2009, 0_12929_02, 0_14221_01, and 0_1688_02) associated with protein kinase family protein, serine-tRNA ligase, and leucine-rich repeat family protein were selected for subsequent sequence amplification and analysis (**Supplementary Table S2**). PCR amplification was conducted in a volume of 25 μL with a DNA concentration of 10–40 ng/μL, 50 mM of Tris-HCl, 0.5 mM of each dNTP, l.5 mM of MgCl_2_, 2 μM of each primer, and 0.75 U of *E*x *Taq* DNA polymerase (Runde, Xi’an, China). The PCR program comprised initial denaturation at 94°C for 5 min, followed by 35 cycles for 1 min at 94°C, at a specific annealing temperature (53–60°C, see **Supplementary Table S2** for details) for 1.5 min and extension for 1 min at 72°C, and a final extension for 10 min at 72°C. Primer synthesis and sequencing of the PCR products were performed by Shanghai Biological Engineering Co. Ltd. (Shanghai, China). Sequencing was conducted using both forward and reverse primers for each gene (**Supplementary Table S2**) on an ABI Prism 3730xl sequencer (Applied Biosystems, Foster City, CA, United States).

### Data Analysis

#### Data Reconciliation

Sequences were aligned and manually adjusted with Chromas and MEGA5.0 (Librado and Rozas, 2009; Tamura et al., 2011) to correct random errors generated by sequencing.

#### Nucleotide Diversity and Neutral Tests

The genetic diversity parameters for the four pine species were calculated using DnaSP v. 5.10 (Librado and Rozas, 2009), including the number of segregation sites *S*, Watterson parameters θ_w_ (Watterson, 1975), total nucleotide polymorphisms π_t_ (Li and Nei, 1975), nucleotide diversities of non-synonymous sites and silent loci (synonymous sites and non-coding positions), π_a_ and π_sil_, number of haplotypes *N*_h_ and haplotype diversity *H*_d_ (Nei and Tajima, 1981; Fu, 1997; Depaulis and Veuille, 1998; Depaulis et al., 2001), and intragenic minimum recombination events (*R*_M_) (Hudson and Kaplan, 1985). In addition, in order to accurately detect departure from the neutral model of molecular evolution at each locus, the neutral equilibrium was tested for various parameters using Tajima’s *D* (Tajima, 1989), Fu and Li’s *D^∗^* and *F^∗^* (Fu and Li, 1993), and the standardized Fay and Wu’s *H* (Fay and Wu, 2000). Tajima’s *D* measures the standardized difference between π and θ_W_, whereas Fay and Wu’s *H* measures the difference between π and θ_H_. The former is more sensitive to an excess of rare variants whereas the latter is more sensitive to an excess of high-frequency-derived variants. Both *D* and *H* are expected to be zero under the standard neutral model (Zou et al., 2013). We also conducted maximum frequency of derived mutations (MFDM) tests to examine the likelihood of natural selection acting on individual loci at species levels. The MFDM tests exclude the confounding effects of demography completely when detecting recent positive selection (Li, 2011). In practice, a single DNA fragment (i.e., a locus) may have a short length and only contain a few *R*_M_. The MFDM v. 1.1 test always depends on the estimate of *R*_M_ (Li, 2011).

#### Genetic Divergence and Population Structure

The sources of genetic variation among the four species (group), populations and individuals were analyzed by analysis of molecular variance (AMOVA) with ARLEQUIN v.3.11 (Excoffier et al., 2005). We estimated *F* statistics hierarchically, both among species (*F*_CT_) and among populations within species (*F*_ST_). *F*_ST_ (Wright, 1949) is a coancestry statistic that provides the variance within populations relative to the total population. We used NETWORK v. 4.6.1.3 (Bandelt et al., 1999) to construct phylogenetic relationships based on the haplotypes of each species at the six loci (gaps were excluded). In addition, genetic clustering based on individuals was estimated by Bayesian clustering using the STRUCTURE V.2.3 program (Hubisz et al., 2009). To estimate the number of clusters (*K*) in the data, *K* values from 1 to 16 were explored using 10 independent runs per *K* and an admixture model. As described in previous studies, in order to generate a reliable estimate of the optimal *K*, the burn-in was set to at least 200,000 and the run length was at least 500,000 (e.g., Zou et al., 2013; Zhou et al., 2014; Tsuda et al., 2015). We also utilized the STRUCTURE HARVESTER program to estimate the most likely number (*K*) of genetic clusters (Evanno et al., 2005; Earl and vonHoldt, 2012).

#### Reconstruction of Historical Dynamics and Species Relationship

The migration rates, effective population sizes, and population split times were calculated based on the isolation-with-migration (IM) model using the IMa2 program to infer the population history dynamics of the four pine species (Nielsen and Wakeley, 2001; Hey, 2006, 2010; Kuhner, 2009). We analyzed sibling species in a pairwise manner using a basic two-population model. We extracted the largest region with no recombination for each of the six nuclear loci. Functions of the model parameters were estimated in the M-mode based on 1 × 10^6^ Monte Carlo Markov chain (MCMC) steps following 5 × 10^5^ burn-in periods in order to obtain reliable estimates (i.e., similar posterior distributions for the parameter), and the effective sample size for each parameter was at least 200. The divergence time between species was estimated based on a mean mutation rate of μ = 4.875 × 10^−9^ (per site per generation), and the generation time for pines was assumed to be 25 years (Ma et al., 2006). In addition, we constructed the phylogenetic relationships among the four pine species using ^∗^BEASTv1.8.0 (Heled and Drummond, 2010). The species tree was computed using the six nuclear genes sequenced for the sampled species. We selected a Yule model as the species tree prior, a constant population size, and relaxed lognormal clock models for all nuclear loci (Heled, 2012). *Pinus bungeana* was used as outgroup. We ran the MCMC analysis for one billion generations with sampling every 50,000 generations. Two independent runs were conducted. Tracer^1^ v1.5 (Rambaut and Drummond, 2009) was used to assess the convergence of chains to the stationary distribution (effective sample size >200). After discarding the first 2,500 trees as a burn-in, the remaining trees were summarized in a maximum clade credibility tree with the TreeAnnotator v1.8.0 program (Drummond and Rambaut, 2007). Joint Bayesian species delimitation and species tree estimation were also analyzed using the BPP v3.4 program based on the multispecies coalescent model (Yang, 2015). In addition, phylogenetic relationships based on nuclear haplotypes were reconstructed with the maximum likelihood (ML) model using PAUP 4.0 (Swofford, 2002). The ML analysis employed the HKY substitution model, where support values for the nodes were estimated based on 1,000 bootstrap replicates.

## Results

### Nucleotide Polymorphisms

For all nuclear loci, *P. griffithii* had the lowest estimates for the total average segregating sites and average values of the segregating sites in silent sites compared with the other three species (**Table 1**). *P. griffithii* had two singleton mutation sites in PTIFG2009. The numbers of shared polymorphisms (*S*_S_) were similar among the four closely related species and the numbers of fixed differences (*S*_f_) were low. The differences in the polymorphisms between *P. pumila* and *P. griffithii* were mainly due to the 0_14221_01 gene locus, with 18 polymorphic sites in *P. pumila* but only three in *P. griffithii* (**Supplementary Table S3**). Similarly, the differences between *P. koraiensis* and *P. pumila* were mainly due to the CL1694 locus, with 20 polymorphic sites in the former but only five in the latter. The 0_14221_01 and 0_12929_02 loci accounted for the observed differences between *P. armandii* and *P. pumila*.

**Table 1:** Nucleotide variations in four *Pinus* species: *Pinus pumila*, *P. griffithii*, *P. koraiensis*, and *P. armandii*.

Species	Locus	Total					Nonsynonymous sites				Silent Sites				*R*m
				
		*N*	L	S (Singl.)	θ	π_*t*_	L	S	θ_*W*_	π_*a*_	L	S	θ_*W*_	π_Sil_	
*P. pumila*	1_1609_01	80	387	14 (0)	0.00850	0.00827	101	3	0.00700	0.00916	282	11	0.00916	0.00807	1
	PTIFG2009	88	505	13 (1)	0.00592	0.00648	194	4	0.00474	0.00600	311	9	0.00666	0.00678	7
	0_1688_02	80	613	6 (0)	0.00233	0.00258	217	0	0.00000	0.00000	387	6	0.00364	0.00404	1
	CL1694	56	305	5 (2)	0.00421	0.00330	59	0	0.00000	0.00000	245	5	0.00524	0.00410	0
	0_12929_02	100	735	14 (1)	0.00425	0.00255	288	2	0.00155	0.00078	443	12	0.00604	0.00372	2
	0_14221_01	68	585	18 (1)	0.00801	0.00779	289	4	0.00391	0.00359	285	15	0.01315	0.01297	4
Average		78		11.67	0.00554	0.00516	191.3	2.2	0.00287	0.00326	325.5	9.7	0.00732	0.00661	15
*P. griffithii*	1_1609_01	56	387	3 (0)	0.00200	0.00162	101	2	0.00509	0.00483	280	1	0.00092	0.00049	0
	PTIFG2009	52	505	9 (2)	0.00467	0.00568	194	3	0.00405	0.00541	311	6	0.00506	0.00585	4
	0_1688_02	52	613	10 (0)	0.00432	0.00291	217	6	0.00726	0.00409	387	4	0.00271	0.00226	0
	CL1694	16	305	0 (0)	0.00000	0.00000	59	0	0.00000	0.00000	245	0	0.00000	0.00000	–
	0_12929_02	40	735	0 (0)	0.00000	0.00000	290	0	0.00000	0.00000	441	0	0.00000	0.00000	–
	0_14221_01	60	585	3 (0)	0.00140	0.00180	289	1	0.00101	0.00174	285	2	0.00178	0.00188	1
Average		46		4.17	0.00207	0.00200	191.7	2	0.00290	0.00268	324.8	2.2	0.00175	0.00175	5
*P. koraiensis*	1_1609_01	24	387	7 (0)	0.00599	0.00775	101	2	0.00659	0.01011	282	5	0.00586	0.00702	0
	PTIFG2009	52	505	11 (2)	0.00571	0.00684	194	2	0.00271	0.00394	311	9	0.00758	0.00865	1
	0_1688_02	20	613	5 (0)	0.00292	0.00374	217	0	0.00000	0.00000	387	5	0.00456	0.00585	1
	CL1694	172	305	19 (0)	0.01305	0.01144	59	2	0.00676	0.00156	245	18	0.01461	0.01386	5
	0_12929_02	20	735	0 (0)	0.00000	0.00000	287	0	0.00000	0.00000	444	0	0.00000	0.00000	–
	0_14221_01	24	539	6 (3)	0.00372	0.00281	289	2	0.00264	0.00187	285	4	0.00477	0.00371	1
Average		52		8.00	0.00523	0.00543	191.2	1.3	0.00312	0.00291	325.7	6.8	0.00623	0.00652	8
*P. armandii*	1_1609_01	80	387	9 (1)	0.00547	0.00519	101	2	0.00467	0.00622	282	7	0.00583	0.00490	0
	PTIFG2009	56	505	12 (4)	0.00611	0.00485	194	3	0.00397	0.00326	311	9	0.00744	0.00584	4
	0_1688_02	96	613	10 (0)	0.00372	0.00661	217	0	0.00000	0.00000	387	10	0.00582	0.01034	2
	CL1694	36	305	6 (0)	0.00572	0.00514	59	1	0.00494	0.00355	245	5	0.00593	0.00555	0
	0_12929_02	52	735	4 (1)	0.00143	0.00212	288	4	0.00364	0.00540	440	0	0.00000	0.00000	0
	0_14221_01	108	539	14 (1)	0.00525	0.00378	293	7	0.00524	0.00380	287	7	0.00536	0.00382	4
Average		71		8.00	0.00462	0.00462	192	2.8	0.00374	0.00371	325.3	6.3	0.00506	0.00508	8

*N, number of individuals; L, length in base pairs; S, number of segregating sites; Singl., number of singleton mutations; θ_W_, Watterson’s parameter (Watterson, 1975); π_t_, nucleotide diversity across all loci; π_a_, nucleotide diversity at nonsynonymous sites; π_sil_, nucleotide diversity at silent sites; Rm, minimum number of recombinant events.*

### Neutrality Tests

Positive Fu and Li’s *D*^∗^ and Fu and Li’s *F*^∗^ values were estimated for most loci, although most of these values were not significant in each species (**Table 2**). The mean Tajima’s *D* (*D*) values were negative for *P. pumila* (−0.236) and *P. griffithii* (−0.030), but positive for *P. koraiensis* (0.274) and *P. armandii* (0.254) (**Table 2**). In addition, the mean Fay and Wu’s *H* (*H*) values were negative for *P. koraiensis* and *P. griffithii* but positive for *P. pumila* and *P. armandii* (**Table 2**). However, with the exception of locus PtIFG2009 (*P* = 0.04674) in *P. pumila*, no significant deviation from neutrality were detected for the six loci using the MFDM test (**Supplementary Table S4**). The MFDM test detected slight deviation from the standard neutral model at the PtIFG2009 locus in the four pine species by considering genetic recombination (*P* < 0.05).

**Table 2:** Haplotype diversity and neutrality tests for *Pinus pumila*, *P. griffithii*, *P. koraiensis*, and *P. armandii*: number of haplotypes (*N*_h_), haplotype diversity (*H*_d_), Tajima’s *D* (*D*), Fu and Li’s *D*^∗^ (*D*^∗^), Fu and Li’s *F*^∗^ (*F*^∗^), and Fay and Wu’s *H* (*H*).

		Haplotype diversity			Neutrality tests		
Species	locus	*N*_h_	*H*_d_	*H*	*D*	*D*^∗^	*F*^∗^
*P. pumila*	1_1609_01	11	0.730	2.503	−0.088	1.535^∗^	1.183
	PTIFG2009	20	0.936	1.801	0.292	1.015	0.916
	0_1688_02	8	0.796	−0.305	0.293	1.192	1.069
	CL1694	6	0.735	0.825	−0.604	−0.576	−0.679
	0_12929_02	12	0.809	1.584	−1.216	1.059	0.346
	0_14221_01	16	0.925	2.624	−0.095	1.288	0.988
Average		12.167	0.822	1.505	−0.236	0.919	0.637
*P. griffithii*	1_1609_01	4	0.550	0.508	−0.456	0.959	0.642
	PTIFG2009	10	0.778	−0.345	0.694	0.253	0.449
	0_1688_02	4	0.551	−0.074	−1.071	1.407^∗^	0.772
	CL1694	1	0.000	−	−	−	−
	0_12929_02	1	0.000	−	−	−	−
	0_14221_01	5	0.641	−0.202	0.654	0.950	1.001
Average		4.167	0.420	−0.019	−0.030	0.432	0.477
*P. koraiensis*	1_1609_01	6	0.818	1.636	1.151	1.351	1.474
	PTIFG2009	7	0.689	−7.409	0.660	0.479	0.623
	0_1688_02	6	0.844	0.533	1.133	1.300	1.411
	CL1694	17	0.820	1.378	−0.363	1.730^∗∗^	1.141
	0_12929_02	1	0.000	−	−	−	−
	0_14221_01	6	0.758	−4.030	−0.934	−0.504	−0.696
Average		7.167	0.655	−1.315	0.274	0.726	0.659
*P. armandii*	1_1609_01	9	0.762	1.554	−0.149	0.733	0.532
	PTIFG2009	13	0.902	−0.138	−0.681	−0.368	−0.543
	0_1688_02	11	0.861	0.051	2.239^∗^	1.399	1.972^∗∗^
	CL1694	6	0.837	0.078	−0.332	1.259	0.943
	0_12929_02	3	0.520	0.019	1.289	0.089	0.501
	0_14221_01	18	0.802	0.122	−0.842	1.051	0.482
Average		10	0.781	0.281	0.254	0.694	0.648

*Significance levels: ^∗^P < 0.05; ^∗∗^P < 0.01.*

### Population Genetic Structure

Within the four species, the 0_12929_02 locus had the highest genetic divergence among populations (*F*_ST_ = 0.714, *P* < 0.001), whereas the 1_1609_01 locus had the lowest genetic divergence among populations (*F*_ST_ = 0.085, *P* < 0.001) (**Supplementary Table S5**). The population genetic divergence was also significant across all loci (*F*_ST_ = 0.624, *P* < 0.001). It should be noted that *F*_ST_ was much higher for the overall loci than interspecific genetic differentiation (*F*_CT_) except for the 0_12929_02 locus (**Supplementary Table S5**). Between pairs of species, *F*_ST_ varied from 0.008 to 1.000 (**Supplementary Table S6**).

The divergence among the four pine species at the six nuclear loci was also supported by Bayesian clustering analysis (**Figure 1**). The most likely number of clusters for the entire dataset was *K* = 3 (**Supplementary Figure S2**). *P. pumila* and *P armandii* individuals were separated into two groups that corresponded to their respective species, whereas the majority of *P. koraiensis* and *P. griffithii* individuals were assigned to another cluster. Remarkable levels of gene flow and gene introgression were apparent between *P. koraiensis* and *P. armandii* (**Figure 1** and **Supplementary Figure S3**).

### Genealogy of Each Locus

**FIGURE 2:**
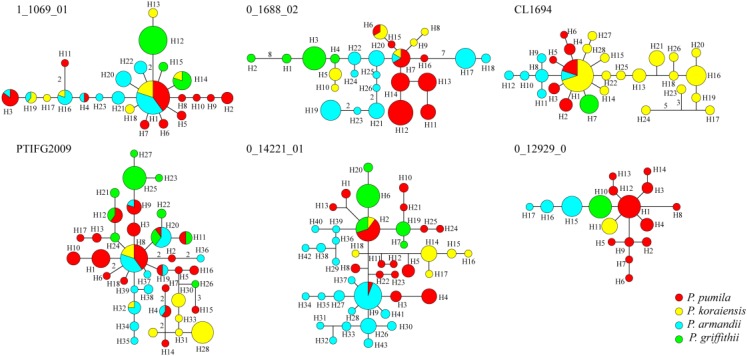
Networks obtained for the six nuclear genes in the four species comprising *Pinus pumila* (*red*), *P. griffithii* (*green*), *P. koraiensis* (*yellow*), and *P. armandii* (*blue*). Each sector of a circle corresponds to the frequency of the haplotype for each species.

The average number of haplotypes (*N*_h_) and haplotype diversity (*H*_d_) were much higher in *P. pumila* (*N*_h_ = 12.167, *H*_d_ = 0.822) and *P. armandii* (*N*_h_ = 10, *H*_d_ = 0.781) than *P. koraiensis* (*N*_h_ = 7.167, *H*_d_ = 0.655) and *P. griffithii* (*N*_h_ = 4.167, *H*_d_ = 0.420) (**Table 2**). The haplotypes in the center of the network were shared (**Figure 2**), but most haplotypes were exclusive to specific species at the five loci (1_1609_01, 0_1688_02, PTIFG2009, 0_14221_01, and CL1694). In addition, there was no shared haplotype at the 0_12929_02 locus. According to the Φ_ST_ values for all loci among the species in **Supplementary Table S7**, each two *Pinus* species exhibited significant genetic divergence, where the highest Φ_ST_ value (0.62006) was found between *P. koraiensis* and *P. griffithii*, whereas the divergence between *P. pumila* and *P. griffithii* was lowest (Φ_ST_ = 0.18941).

### Evolutionary Relationships Among the Four Species

**FIGURE 3:**
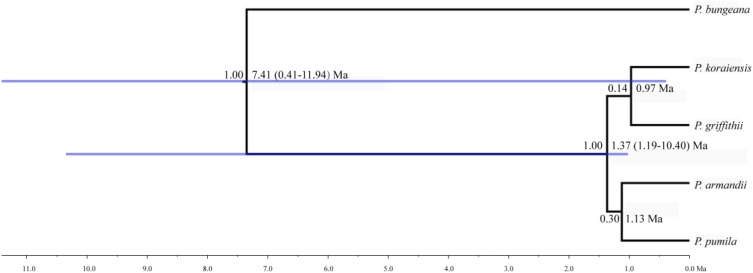
Divergence times and phylogenetic relationships among four pine species. The tree was constructed based on six nuclear genes using ^∗^BEAST. The tree was rooted with *Pinus bungeana*. The numbers on the branches indicate the corresponding posterior probabilities values, mean divergence dates, and 95% credibility interval.

The mean divergence time between *P. pumila* and *P. armandii* was estimated at 1.13 million years ago (Mya). A younger divergence time (0.319 Mya) was estimated between *P. griffithii* and *P. koraiensis* (**Table 3**). In addition, we found asymmetric historical gene flow between pairs of species. In particular, the migration rate from *P. pumila* to *P. griffithii* was 2.0450, with 0.0005 in the reverse direction (**Table 3**). *Pinus griffithii* and *P. koraiensis* had smaller population sizes (0.0918–0.1846 and 0.3264–0.5369, respectively; **Table 3**) than *P. pumila* and *P. armandii* (0.3738–0.8326 and 0.4087–0.7019, respectively; **Table 3**). The species tree analyses demonstrated that the relationship was closer between *P. armandii* and *P. pumila* where they clustered as sister groups, whereas *P. koraiensis* and *P. griffithii* were located in another clade. The four species split into two groups about 1.37 Mya (**Figure 3**). Moreover, we obtained the best species-tree model using BPP v3.4 software, where the posterior probability of the species tree was one and the acceptance proportion was near to zero (0.025) based on multiple runs (**Supplementary Figure S4**). The topology of the tree was consistent with the results obtained by ^∗^BEAST (**Figure 3** and **Supplementary Figure S4**).

**Table 3:** Maximum-likelihood estimates and 90% highest posterior density (HPD) intervals for demographic parameters obtained from pairwise IM multilocus analyses.

Comparison	*q*_1_	*q*_2_	*q*_a_	*m*_1_	*m*_2_	*t*	*T* (Mya)
*P. pumila* and *P. griffithii*	0.8326	0.0918	2.8650	0.0005	2.0450	0.9050	0.356
HPD90Lo	0.5481	0.0407	0.7525	0.0005	0.7850	0.4850	0.191
HPD90Hi	1.1916	0.1898	12.9304	0.4605	5.1450	9.1150	3.582
*P. pumila* and *P. koraiensis*	0.8142	0.3264	4.8591	0.2275	1.8865	1.9190	0.754
HPD90Lo	0.5571	0.1800	1.3648	0.0025	0.0035	1.0450	0.411
HPD90Hi	1.1505	0.5637	13.1795	1.6375	3.8465	33.9150	13.327
*P. pumila* and *P. armandii*	0.3738	0.4087	0.5456	0.4550	3.1950	2.6200	1.030
HPD90Lo	0.1840	0.2111	0.0032	0.0050	0.5650	1.3400	0.527
HPD90Hi	0.6528	0.7457	5.8013	3.9750	6.9750	39.9800	15.711
*P. griffithii* and *P. koraiensis*	0.1470	0.5369	1.5049	1.0750	0.2950	0.8125	0.319
HPD90Lo	0.0624	0.2989	0.6903	0.0350	0.0050	0.3175	0.125
HPD90Hi	0.3057	0.9019	5.2869	3.5450	1.7350	4.5675	1.795
*P. griffithii* and *P. armandii*	0.1846	0.7019	1.4730	1.1525	0.3050	5.6875	2.235
HPD90Lo	0.0726	0.4046	0.0346	0.0575	0.0050	1.7875	0.702
HPD90Hi	0.4087	1.1445	12.4550	3.5175	1.8650	64.8375	25.479
*P. koraiensis* and *P. armandii*	0.4364	0.6272	0.2650	0.4305	0.0100	5.7900	2.275
HPD90Lo	0.2209	0.3327	0.0059	0.0035	0.0100	1.8700	0.735
HPD90Hi	0.8251	1.0748	10.5714	5.0505	4.4100	18.7300	7.360

*q_1_, q_2_, and q_a_, parameters of population sizes of the first and second species and their ancestral population, respectively; t, parameter of the divergence time between two species; m_1_ is the population migration rate from the second to the first species, m_2_ is the population migration rate from the first to the second species; T is the divergence time among two species. HPD, 90% posterior density (90% low and 90% high).*

## Discussion

### Nucleotide Diversity

The nucleotide diversity at silent sites basically agreed with the neutral model of molecular evolution (Ma et al., 2006; Wachowiak et al., 2016). We detected low levels of silent polymorphisms in the four closely related pines species, because the average values were much lower than the average polymorphism for most conifers at multiple nuclear genes (π_sil_ = 0.0029–0.0122) (Ma et al., 2006; Li et al., 2012). Among the four species, *P. pumila* had the highest silent nucleotide diversity (π_sil_ = 0.00661), whereas *P. griffithii* had the lowest (π_sil_ = 0.00175). However, these diversity values were much lower than those found in other *Pinus* species, such as *P. densata* and *P. yunnanensis* (Ma et al., 2006). Factors such as the nuclear gene loci selected in the study, sample size variations, mutation rates within species, demographic effects, and natural selection can influence the nucleotide diversity levels and patterns in species or populations (Loveless and Hamrick, 1984; Lande, 1988; Li et al., 2012; Zou et al., 2013). The four related species and six nuclear loci investigated in our research have also been studied previously, and thus these nuclear loci were not the main cause of the low levels of nucleotide polymorphisms. However, the unequal sample sizes for different genes and species may have caused differences in the nucleotide variability among species. To verify this bias, we detected the nucleotide diversity parameters based on the same sample sizes for each gene from each *Pinus* species. The results showed that there were significant differences in the levels of diversity among different species compared with the previous estimates (**Table 1** and **Supplementary Table S8**). We concluded that the levels of nucleotide variability among species were significantly associated with the samples sizes of the pine species. Similar differences in the patterns of diversity have also been detected in some other gymnosperm species (Ma et al., 2006; Du et al., 2009; Zou et al., 2013; Zhou et al., 2014). In addition, the long life cycles and low mutation rates in conifers may explain the low levels of nucleotide polymorphisms in *P. pumila*, *P. griffithii*, *P. koraiensis*, and *P. armandii.* Moreover, high levels of linkage disequilibrium were detected and some species deviated from neutrality according to the tests conducted in our study. This was particularly evident at one locus, i.e., PtIFG2009, which suggests that this locus might have undergone selection or population shrinkage according to the results obtained from the Tajima’s *D* and MFDM tests. In particular, for the populations of *P. griffithii* and *P. koraiensis*, Tajima’s *D* was positive, and Fay and Wu’s *H* was negative, which is a pattern that is consistent with a recent bottleneck. In addition, *P. griffithii* and *P. pumila* descendant populations had a somewhat smaller size than the ancestral population (**Table 3**), and thus it is possible that the populations have experienced from genetic bottlenecks. The population dynamics due to geological isolation and climatic oscillations probably contributed to their relatively lower diversity (Chen et al., 2017). In addition, the mean Tajima’s D (*D*) and mean Fay and Wu’s H (*H*) values were negative but close to zero for *P. armandii* at the PtIFG2009 locus, which may indicate a neutral equilibrium (Holliday et al., 2010). The numbers of nucleotide polymorphism were higher in *P. pumila*, *P. koraiensis*, and *P. armandii* than *P. griffithii*. These results can partly be explained by the fact that their seeds are food for nutcrackers and rodents such as squirrels. These animals may screen the seeds and transport them over long or short distances for secondary storage and dispersal, thereby also enhancing the spread of the seeds, and this may affect their genetic differences (Li et al., 2007; Fan and Jin, 2011). The low level of nucleotide polymorphism in *P. griffithii* may be explained by its small geographic distribution compared with more common and widespread species, because of drift, founder events, and other stochastic processes (Cole, 2003; Chen et al., 2017). In addition, *P. pumila* is well known because of its larger island-like distribution and it rarely develops into pure forest, thereby accounting for its higher diversity compared with the other three species (Qiu et al., 2007; Chen et al., 2017).

### Interspecific Gene Flow and Species Divergence

Analysis of molecular variance detected remarkable divergence in the four *Pinus* species where the variations were mainly within populations, which agreed with the small differences among populations of wind-pollinated gymnosperms (**Supplementary Table S5**). However, we also found a high level of genetic divergence within groups, possibly due to habitat fragmentation or the island-like distribution of the four species, particularly when considering that the habitats of *P. pumila* and *P. griffithii* are harsher than those of the other two species. The former often grows in barren soil on bare rocky peaks. This type of habitat is vulnerable to fragmentation but mountains and ravines may partly hinder the gene flow between populations, thereby leading to the isolation of groups. Genetic divergence was found among the four species, although some degree of gene flow and introgression was detected. In particular, populations of *P. koraiensis* had a mosaic-like pattern and they were further subdivided into independent sub-clusters when *K* = 4, which suggests that a high level of introgression in this species. The pairwise migration rate between *P. koraiensis* and *P. pumila* was relatively high compared with that between *P. koraiensis* and *P. armandii*. In addition, *P. pumila* and *P. koraiensis* had a relatively limited distribution in the northwest and northeast of North China, and there was no clear phylogenetic resolution among *P. pumila*, *P. koraiensis*, and *P. armandii* based on DNA fragments from the chloroplast, mitochondrial, and nuclear genomes according to previous phylogenetic studies (Liu et al., 2014; Wang and Wang, 2014; Hao et al., 2015). These results suggest that migration may have led to a sympatric distribution in addition to the existing incomplete reproductive isolation. The phylogenetic relationships determined based on ML and NETWORK analysis also showed that the shared haplotypes were located in the center of the topological structure (**Figure 2** and **Supplementary Figure S5**), and thus incomplete genealogical screening based on a large effective population of *Pinus* may have led to the sharing of ancestral polymorphisms. However, there were no shared haplotypes based on the 0_12929_02 locus, and the interspecies variation was similar to the genetic differentiation among populations (*F*_CT_ = 0.718, *F*_ST_ = 0.714; *P* < 0.001; **Supplementary Table S5**). In general, different nuclear loci have different evolutionary rates and molecular functions (Li et al., 2012; Eckert et al., 2013; Zhou et al., 2014). Previous studies have shown that the 0_12929_02 locus is associated with the protein kinase family and that it has been under selection (Eckert et al., 2013). The rapid fixation of genetic variation in this locus may have led to greater species divergence (Nei et al., 1975; Nei and Tajima, 1981; Ellstrand and Elam, 1993). In addition, nuclear DNA introgression in ancestral populations among species may have also affected the topology of the phylogenetic trees. The significant topological incongruence among the nuclear gene trees (**Supplementary Figure S5**) indicates a complex evolutionary history, thereby providing novel insights into the evolution of *Pinus*. The four species split into two clades about 1.37 Mya (**Figure 3**). However, we should be cautious when inferring divergence times based on the assumption that the generation time is 25 years in the four *Pinus* species because of the longevity of *Pinus*, the long overlaps between generations, the variable age of maturity and the replacement speed of forests. In addition, our multilocus analysis determined that the genetic divergence among the four pine species, was consistent with geological events and climatic oscillations in the mid- to late Tertiary period about 5 Mya. The uplift of the Tibetan Plateau caused by Himalayan orogeny had a great impact on the climate in China, with decreases of in temperature in some areas, but increases of 4–8°C in the region east of 100°E (across the Inner Mongolia, Gansu, Qinghai, Sichuan, and Yunnan regions of China) and of 1–4°C to the west (Jiang, 2009). These climatic conditions may have changed the geographic distributions of plants, and thus we suggest that *P. armandii* and its ancestral population spread eastward to the northeast of China and westward to Tibet. However, the intensities of the winter and summer monsoons were reduced greatly during the middle-late Pliocene, and the dispersal of Pinaceae pollen by the wind might have been affected (Zhou et al., 2014). Moreover, after gradual changes in the microhabitats and variations in the directions and amounts of gene flow, as well as the accumulation of mutations, new relatives may have emerged by gradual divergence. Effective migration, hybridization, and introgression among species can increase genetic diversity (Wachowiak et al., 2016), and other factors such as selection, isolation, and genetic drift among different microhabitats can promote divergence and speciation. Indeed, significant and asymmetric gene flow and introgression were detected in the four closely related *Pinus* species. Gene flow and genetic introgression among different pines could have led to changes in genetic variability (Hao et al., 2015; Wachowiak et al., 2016).

## Data Availability

Sequence data obtained in this study were deposited in GenBank (KF286539 – KF286739).

## Author Contributions

Z-HL conceived the study. YJ and JZ performed the experiments. Z-HL, YJ, YW, W-BF, and G-FZ contributed materials and analysis tools. Z-HL, YJ, and JZ wrote the manuscript. YJ and ZL revised the manuscript. All authors approved the final version of the manuscript.

## Conflict of Interest Statement

The authors declare that the research was conducted in the absence of any commercial or financial relationships that could be construed as a potential conflict of interest.

## Footnotes

1 http://tree.bio.ed.ac.uk/software/tracer/

## Supplementary Material

FIGURE S1 The occurrence records were denoted by small dots of different colors for four related pine species: *Pinus pumila* (*red*), *P. griffithii* (*green*), *P. koraiensis* (*yellow*), and *P. armandii* (*blue*). The large dots of four different colors represent the current sampling locations for four pines.

FIGURE S2 Bayesian inference analysis of nuclear data to determine the most likely number of clusters (*K*) for the four pine species. Distributions of the likelihood L (*K*) values **(A)** and delta *K* values **(B)** are presented for *K* = 1–16.

FIGURE S3 Probability of assignments of four closely related pine species into two and four ancestral clusters (*K* = 2 and *K* = 4) estimated by the STRUCTURE program.

FIGURE S4 Dendrogram derived for four closely related pines species using BPP with six nuclear loci sequences. Bootstrap values are shown above each branch in the BPP tree.

FIGURE S5 Maximum likelihood (ML) tree based on the nuclear haplotypes built using PAUP version 4.0b10. *Pinus bungeana* was designated as an outgroup. Bootstrap values for ML analyses are shown above branch in the trees. Pie charts indicate the probabilities of the haplotypes for each species.

TABLE S1 Sampling locality and altitude for *Pinus pumila*, *P. griffithii*, *P. koraiensis*, and *P. armandii*.

TABLE S2 Details of the primers used in this study.

TABLE S3 Distribution of polymorphic sites in the four related pine species: *Pinus pumila*, *P. griffithii*, *P. koraiensis*, and *P. armandii*. *S*_1_, number of exclusive polymorphic sites in the first species; *S*_2_, number of exclusive polymorphic sites in the second species; *S*_S_, number of shared polymorphisms; *S*_f_, number of fixed differences between two species.

TABLE S4 Maximum frequency of derived mutations (MFDM) test results for the four pine species.

TABLE S5 Analysis of molecular variance (AMOVA) for nucleotide sequence variations in four *Pinus* species.

TABLE S6 Genetic divergence (*F*_ST_) at each locus among specie based on pairwise comparisons for *P. armandii*, *P. griffithii*, *P. koraiensis* and *P. pumila*.

TABLE S7 Φ_ST_ values over all loci among species.

TABLE S8 Nucleotide variations in *Pinus pumila*, *P. griffithii*, *P. koraiensis*, and *P. armandii* with equal sample sizes.

## References

[B1] Bandelt H. J. Forster P. Rohl A. (1999). Median-joining networks for inferring intraspecific phylogenies. *Mol. Biol. Evol.* 16 37–48. 10.1093/oxfordjournals.molbev.a02603 10331250

[B2] Chen C. Lu R. S. Zhu S. S. Tamaki I. Qiu Y. X. (2017). Population structure and historical demography of *Dipteronia dyeriana* (Sapindaceae), an extremely narrow palaeoendemic plant from china: implications for conservation in a biodiversity hot spot. *Heredity* 119 95–106. 10.1038/hdy.2017.19 28379211 PMC5520545

[B3] Chen J. Källman T. Gyllenstrand N. Lascoux M. (2010). New insights on the speciation history and nucleotide diversity of three boreal spruce species and a Tertiary relict. *Heredity* 104 3–14. 10.1038/hdy.2009.88 19639012

[B4] Cole C. T. (2003). Genetic variation in rare and common plants. *Annu. Rev. Ecol. Evol. Syst.* 2003 213–237. 10.1146/annurev.ecolsys.34.030102.151717

[B5] Coyne J. A. Orr H. A. (2004). *Speciation.* Sunderland, MA: Sinauer Associates, Inc.

[B6] Cutter A. D. Gray J. C. (2016). Ephemeral ecological speciation and the latitudinal biodiversity gradient. *Evolution* 70 2171–2185. 10.1111/evo.13030 27502055

[B7] Depaulis F. Mousset S. Veuille M. (2001). Haplotype tests using coalescent simulations conditional on the number of segregating sites. *Mol. Biol. Evol.* 18 1136–1138. 10.1093/oxfordjournals.molbev.a003885 11371602

[B8] Depaulis F. Veuille M. (1998). Neutrality tests based on the distribution of haplotypes under an infinite-site model. *Mol. Biol. Evol.* 15 1788–1790. 10.1093/oxfordjournals.molbev.a025905 9917213

[B9] Doyle J. J. Doyle J. L. (1987). A rapid DNA isolation procedure for small quantities of fresh leaf tissue. *Phytochem. Bull.* 19 11–15.

[B10] Drummond A. J. Rambaut A. (2007). BEAST: bayesian evolutionary analysis by sampling trees. *BMC Evol. Biol.* 7:214. 10.1186/1471-2148-7-214 17996036 PMC2247476

[B11] Du F. K. Petit R. J. Liu J. Q. (2009). More introgression with less gene flow: chloroplast vs. mitochondrial DNA in the *Picea asperata* complex in China, and comparison with other conifers. *Mol. Ecol.* 18 1396–1407. 10.1111/j.1365-294X.2009.04107.x 19284474

[B12] Earl D. vonHoldt B. (2012). STRUCTURE HARVESTER: a website and program for visualizing STRUCTURE output and implementing the Evanno method. *Conserv. Genet. Resour.* 4 359–361. 10.1007/s12686-011-9548-7

[B13] Eckert A. J. Bower A. D. Jermstad K. D. Wegrzyn J. L. Knaus B. J. Syring J. V. (2013). Multilocus analyses reveal little evidence for lineage-wide adaptive evolution within major clades of soft pines (*Pinus* subgenus *Strobus*). *Mol. Ecol.* 22 5635–5650. 10.1111/mec.12514 24134614

[B14] Ellstrand N. C. Elam D. R. (1993). Population genetic consequences of small population size: implications for plant conservation. *Annu. Rev. Ecol. Syst.* 24 217–242. 10.1146/annurev.es.24.110193.001245

[B15] Evanno G. Regnaut S. Goudet J. (2005). Detecting the number of clusters of individuals using the software structure: a simulation study. *Mol. Ecol.* 14 2611–2620. 10.1111/j.1365-294X.2005.02553.x 15969739

[B16] Excoffier L. Laval G. Schneider S. (2005). Arlequin (version 3.0): an integrated software package for population genetics data analysis. *Evol. Bioinform. Online* 1 47–50. 10.1177/117693430500100003 19325852 PMC2658868

[B17] Fan C. Jin C. (2011). Effects of *P. armandii* seed size on rodents caching behavior and it’s spatio-temporal variations. *Zool. Res.* 32 435–441. 10.3724/SP.J.1141.2011.04435 21842540

[B18] Fay J. C. Wu C. I. (2000). Hitchhiking under positive Darwinian selection. *Genetics* 155 1405–1413. 10880498 10.1093/genetics/155.3.1405 PMC1461156

[B19] Fu L. G. Li N. Mill R. R. (1999). “Pinaceae,” in *Flora of China* Vol. 4 eds Wu Z. Y. Raven P. H. (Beijing: Science Press), 11–52.

[B20] Fu Y. X. (1997). Statistical tests of neutrality of mutations against population growth, hitchhiking and background selection. *Genetics* 147 915–925. 9335623 10.1093/genetics/147.2.915 PMC1208208

[B21] Fu Y. X. Li W. H. (1993). Statistical tests of neutrality of mutations. *Genetics* 133 693–709. 8454210 10.1093/genetics/133.3.693 PMC1205353

[B22] Gao J. Wang B. S. Mao I. F. Ingvarsson P. Zeng Q. Y. Wang X. R. (2012). Demography and speciation history of the homoploid hybrid pine *Pinus densata* on the Tibetan Plateau. *Mol. Ecol.* 21 4811–4827. 10.1111/j.1365-294X.2012.05712.x 22849551

[B23] Hao Z. Z. Liu Y. Y. Nazaire M. Wei X. X. Wang X. Q. (2015). Molecular phylogenetics and evolutionary history of sect. Quinquefoliae (*Pinus*): implications for Northern Hemisphere biogeography. *Mol. Phylogenet. Evol.* 87 65–79. 10.1016/j.ympev.2015.03.013 25800283

[B24] Heled J. (2012). Sequence diversity under the multispecies coalescent with Yule process and constant population size. *Theor. Popul. Biol.* 81 97–101. 10.1016/j.tpb.2011.12.007 22210390

[B25] Heled J. Drummond A. J. (2010). Bayesian inference of species trees from multilocus data. *Mol. Biol. Evol.* 27 570–580. 10.1093/molbev/msp274 19906793 PMC2822290

[B26] Hey J. (2006). Recent advances in assessing gene flow between diverging populations and species. *Curr. Opin. Genet. Dev.* 16 592–596. 10.1016/j.gde.2006.10.005 17055250

[B27] Hey J. (2010). Isolation with migration models for more than two populations. *Mol. Biol. Evol.* 27 905–920. 10.1093/molbev/msp296 19955477 PMC2877539

[B28] Holliday J. A. Yuen M. Ritland K. Aitken S. N. (2010). Postglacial history of a widespread conifer produces inverse clines in selective neutrality tests. *Mol. Ecol.* 19 3857–3864. 10.1111/j.1365-294X.2010.04767.x 20738783

[B29] Hubisz M. J. Falush D. Stephens M. Pritchard J. K. (2009). Inferring weak population structure with the assistance of sample group information. *Mol. Ecol. Resour.* 9 1322–1332. 10.1111/j.1755-0998.2009.02591.x 21564903 PMC3518025

[B30] Hudson R. R. Kaplan N. L. (1985). Statistical properties of the number of recombination events in the history of a sample of DNA sequences. *Genetics* 111 147–164. 4029609 10.1093/genetics/111.1.147 PMC1202594

[B31] Jiang D. B. (2009). Numerical simulation analysis of Chinese climate in middle Pliocene. *Quat. Res.* 29 1033–1043.

[B32] Kuhner M. K. (2009). Coalescent genealogy samplers: windows into population history. *Trens. Ecol. Evol.* 24 86–93. 10.1016/j.tree.2008.09.007 19101058 PMC4714702

[B33] Lande R. (1988). Genetics and demography in biological conservation. *Science* 241 1455–1460. 10.1126/science.3420403 3420403

[B34] Li H. J. Ma J. Z. Zong C. (2007). Compare of behaviors of four kinds of diurnal animals about feeding and storage of *Pinus koraiensis* seeds. *Chin. J. Zool.* 42 10–16.

[B35] Li H. P. (2011). A new test for detecting recent positive selection that is free from the confounding impacts of demography. *Mol. Biol. Evol.* 28 365–375. 10.1093/molbev/msq211 20709734

[B36] Li L. Abbott R. J. Liu B. Sun Y. Li L. (2013). Pliocene intraspecific divergence and Plio-Pleistocene range expansions within *Picea likiangensis* (Lijiang spruce), a dominant forest tree of the Qinghai-Tibet Plateau. *Mol. Ecol.* 22 5237–5255. 10.1111/mec.12466 24118118

[B37] Li W. H. Nei M. (1975). Drift variances of heterozygosity and genetic distance in transient states. *Genet. Res.* 25 229–247. 10.1017/S0016672300015664 1183809

[B38] Li Y. Stocks M. Hemmilä S. Källman T. Zhu H. T. Zhou Y. F. (2010). Demographic histories of four spruce (*Picea*) species of the Qinghai-Tibetan Plateau and neighboring areas inferred from multiple nuclear loci. *Mol. Biol. Evol.* 27 1001–1014. 10.1093/molbev/msp301 20031927

[B39] Li Z. H. Yang C. Mao K. S. Ma Y. Z. Liu J. Liu Z. L. (2015). Molecular identification and allopatric divergence of the white pine species in China based on the cytoplasmic DNA variation. *Biochem. Syst. Ecol.* 61 161–168. 10.1016/j.bse.2015.06.002

[B40] Li Z. H. Zou J. B. Mao K. S. Lin K. Li H. P. Liu J. Q. (2012). Population genetic evidence for complex evolutionary histories of four high altitude juniper species in the Qinghai-Tibetan Plateau. *Evolution* 66 831–845. 10.1111/j.1558-5646.2011.01466.x 22380443

[B41] Librado P. Rozas J. (2009). DnaSP v5: a software for comprehensive analysis of DNA polymorphism data. *Bioinformatics* 25 1451–1452. 10.1093/bioinformatics/btp187 19346325

[B42] Liu J. Hao Z. Z. Liu Y. Y. Wei X. Z. Cun Y. Z. Wang X. Q. (2014). Phylogeography of *Pinus armandii* and its relatives: heterogeneous contributions of geography and climate changes to the genetic differentiation and diversification of Chinese white pines. *PLoS One* 9:e85920. 10.1371/journal.pone.0085920 24465789 PMC3897548

[B43] Loveless M. D. Hamrick J. L. (1984). Ecological determinants of genetic structure in plant populations. *Annu. Rev. Ecol. Syst.* 15 65–95. 10.1146/annurev.es.15.110184.000433

[B44] Ma X. F. Szmidt A. E. Wang X. R. (2006). Genetic structure and evolutionary history of a diploid hybrid pine *Pinus densata* inferred from the nucleotide variation at seven gene loci. *Mol. Biol. Evol.* 23 807–816. 10.1093/molbev/msj100 16446291

[B45] Neale D. B. Kremer A. (2011). Forest tree genomics: growing resources and applications. *Nat. Rev. Genet.* 12 111–122. 10.1038/nrg2931 21245829

[B46] Nei M. Maruyama T. Chakraborty R. (1975). The bottleneck effect and genetic variability in populations. *Evolution* 29 1–10. 10.1111/j.1558-5646.1975.tb00807.x 28563291

[B47] Nei M. Tajima F. (1981). DNA polymorphism detectable by restriction endonucleases. *Genetics* 97 145–163. 6266912 10.1093/genetics/97.1.145 PMC1214380

[B48] Nielsen R. Wakeley J. (2001). Distinguishing migration from isolation: a Markov chain Monte Carlo approach. *Genetics* 158 885–896. 11404349 10.1093/genetics/158.2.885 PMC1461674

[B49] Ortego J. Noguerales V. Gugger P. F. Sork V. L. (2015). Evolutionary and demographic history of the Californian scrub white oak species complex: an integrative approach. *Mol. Ecol.* 24 6188–6208. 10.1111/mec.13457 26547661

[B50] Peng Z. H. (1999). *Pinus* sect, strobus originated in China. *J. Anhui Agric.* 26 1–8.

[B51] Qiu Y. X. Luo Y. P. Comes H. P. Ouyang Z. Q. Fu C. X. (2007). Population genetic diversity and structure of *Dipteronia dyerana* (Sapindaceae), a rare endemic from Yunnan province, China, with implications for conservation. *Taxon* 56 427–437.

[B52] Rambaut A. Drummond A. J. (2009). *Tracer v1.5.* Available at: http://tree.bio.ed.ac.uk/software/tracer/

[B53] Ren G. Mateo R. G. Liu J. Suchan T. Alvarez N. Guisan A. (2017). Genetic consequences of quaternary climatic oscillations in the Himalayas: *Primula tibetica* as a case study based on restriction site-associated DNA sequencing. *New Phytol.* 213 1500–1512. 10.1111/nph.14221 27696413

[B54] Ren G. P. Abbott R. J. Zhou Y. F. Zhang L. R. Peng Y. L. Liu J. Q. (2012). Genetic divergence, range expansion and possible homoploid hybrid speciation among pine species in northeast China. *Heredity* 108 552–562. 10.1038/hdy.2011.123 22187083 PMC3330684

[B55] Swofford D. L. (2002). *PAUP^∗^4.0: Phylogenetic Analysis Using Parsimony*. Sunderland, MA: Sinauer Associates.

[B56] Syring J. Farrell K. Businskı R. Cronn R. Liston A. (2007). Widespread genealogical nonmonophyly in species of *Pinus* subgenus *Strobus*. *Syst. Biol.* 56 163–181. 10.1080/10635150701258787 17454973

[B57] Tajima F. (1989). The effect of change in population size on DNA polymorphism. *Genetics* 123 597–601. 2599369 10.1093/genetics/123.3.597 PMC1203832

[B58] Tamura K. Peterson D. Peterson N. Stecher G. Nei M. Kumar S. (2011). MEGA5: molecular evolutionary genetics analysis using maximum likelihood, evolutionary distance, and maximum parsimony methods. *Mol. Biol. Evol.* 28 2731–2739. 10.1093/molbev/msr121 21546353 PMC3203626

[B59] Tsuda Y. Nakao K. Ide Y. Tsumura Y. (2015). The population demography of *Betula maximowicziana*, a cool-temperate tree species in Japan, in relation to the last glacial period: its admixture-like genetic structure is the result of simple population splitting not admixing. *Mol. Ecol.* 24 1403–1418. 10.1111/mec.13123 25706115

[B60] Tsuda Y. Semerikov V. Sebastiani F. Vendramin G. G. Lascoux M. (2017). Multispecies genetic structure and hybridization in the *Betula* genus across Eurasia. *Mol. Ecol.* 26 589–605. 10.1111/mec.13885 27763698

[B61] Wachowiak W. Balk P. Savolainen O. (2009). Search for nucleotide diversity patterns of local adaptation in dehydrins and other cold-related candidate genes in Scots pine (*Pinus sylvestris* L.). *Tree Genet. Genomes* 5 117–132. 10.1007/s11295-008-0188-3

[B62] Wachowiak W. Boratyńska K. Cavers S. (2013). Geographical patterns of nucleotide diversity and population differentiation in three closely related European pine species in the *Pinus mugo* complex. *Bot. J. Linn. Soc.* 172 225–238. 10.1111/boj.12049

[B63] Wachowiak W. Żukowska W. B. Wójkiewicz B. Cavers S. Litkowiec M. (2016). Hybridization in contact zone between temperate European pine species. *Tree Genet. Genomes* 12:48 10.1007/s11295-016-1007-x

[B64] Wang B. Wang X. R. (2014). Mitochondrial DNA capture and divergence in *Pinus* provide new insights into the evolution of the genus. *Mol. Phylogenet. Evol.* 80 20–30. 10.1016/j.ympev.2014.07.014 25106134

[B65] Wang J. Street N. R. Scofield D. G. Ingvarsson P. K. (2016). Natural selection and recombination rate variation shape nucleotide polymorphism across the genomes of three related *Populus* species. *Genetics* 202 1185–1200. 10.1534/genetics.115.183152 26721855 PMC4788117

[B66] Watterson G. A. (1975). On the number of segregating sites in genetical models without recombination. *Theor. Popul. Biol.* 7 256–276. 10.1016/0040-5809(75)90020-9 1145509

[B67] Willyard A. Cronn R. Liston A. (2009). Reticulate evolution and incomplete lineage sorting among the ponderosa pines. *Mol. Phylogenet. Evol.* 52 498–511. 10.1016/j.ympev.2009.02.011 19249377

[B68] Wright S. (1949). The genetical structure of populations. *Ann. Hum. Genet.* 15 323–354. 10.1111/j.1469-1809.1949.tb02451.x 24540312

[B69] Wright S. I. Gaut B. S. (2005). Molecular population genetics and the search for adaptive evolution in plants. *Mol. Biol. Evol.* 22 506–519. 10.1093/molbev/msi035 15525701

[B70] Wu G. Feng Z. W. (1995). Research about community characteristics and biomass of *Pinus* sect, strobus in China. *Acta Ecol. Sin.* 15 260–267.

[B71] Yang Z. (2015). The BPP program for species tree estimation and species delimitation. *Curr. Zool.* 61 854–865. 10.1093/sysbio/syy051 29982825 PMC6292489

[B72] Zhou Y. F. Zhang L. R. Liu J. Q. Wu G. L. Savolainen O. (2014). Climatic adaptation and ecological divergence between two closely related pine species in Southeast China. *Mol. Ecol.* 23 3504–3522. 10.1111/mec.12830 24935279

[B73] Zou J. B. Sun Y. S. Li L. Wang G. N. Yue W. Lu Z. Q. (2013). Population genetic evidence for speciation pattern and gene flow between *Picea wilsonii*, *P. morrisonicola* and *P. neoveitchii*. *Ann. Bot.* 112 1829–1844. 10.1093/aob/mct241 24220103 PMC3838563

[B74] Zou J. B. Yue W. Li L. L. Wang X. Lu J. Duan B. B. (2016). DNA barcoding of recently diversified tree species: a case study on spruces based on 20 DNA fragments from three different genomes. *Trees Struct. Funct.* 30 959–969. 10.1007/s00468-015-1337-6

