# Supplementary figures and images for: Effects of Geological and Environmental Events on the Diversity and Genetic Divergence of Four Closely Related Pines: Pinus koraiensis, P. armandii, P. griffithii, and P. pumila
Source: Front Plant Sci. 2018 Aug 28;9:1264. doi: 10.3389/fpls.2018.01264 (PMC6121107; doi:10.3389/fpls.2018.01264)

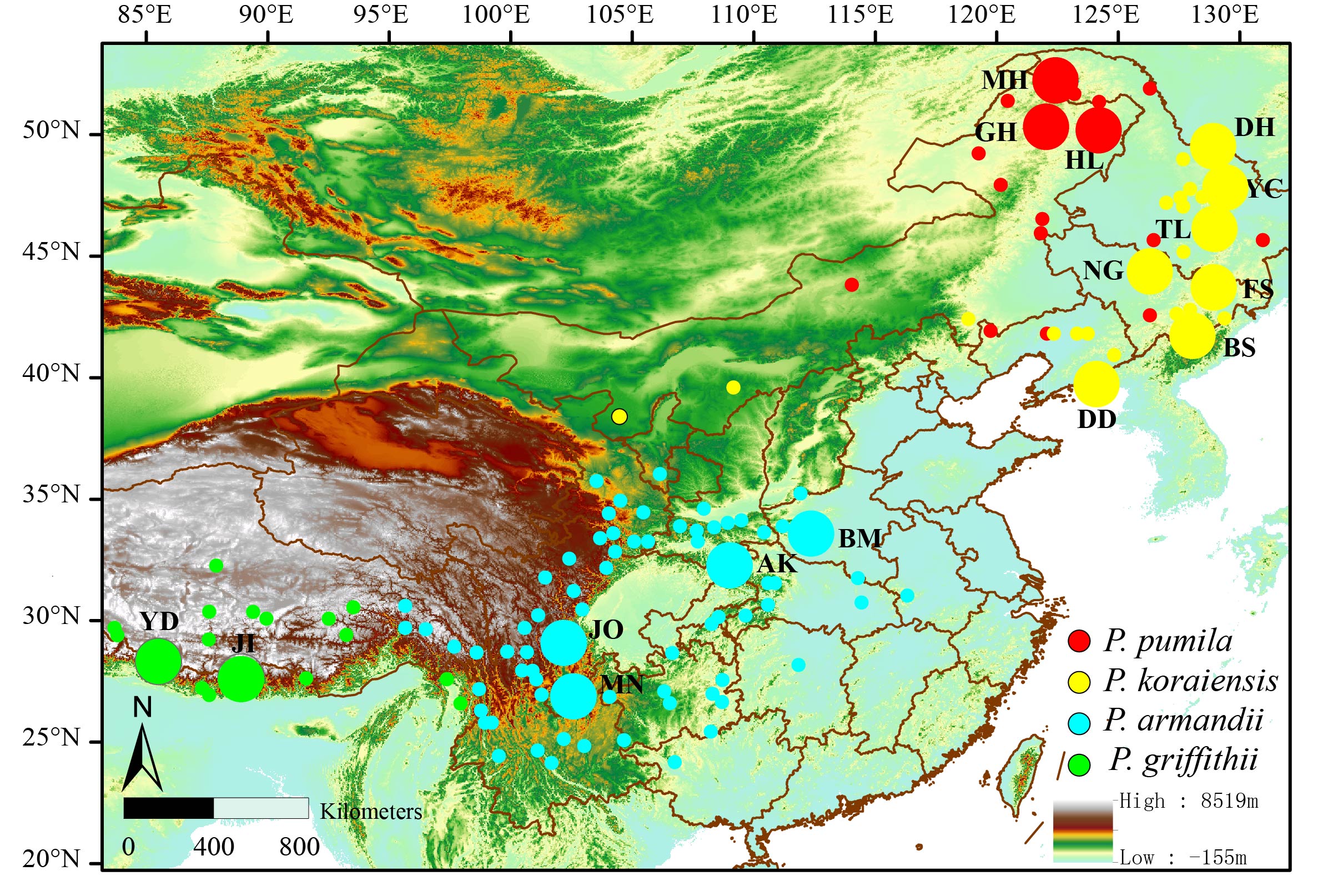

Supplement: FIGURE S1 — The occurrence records were denoted by small dots of different colors for four related pine species: Pinus pumila (red), P. griffithii (green), P. koraiensis (yellow), and P. armandii (blue). The large dots of four different colors represent the current sampling locations for four pines. [file Image_1.JPEG]

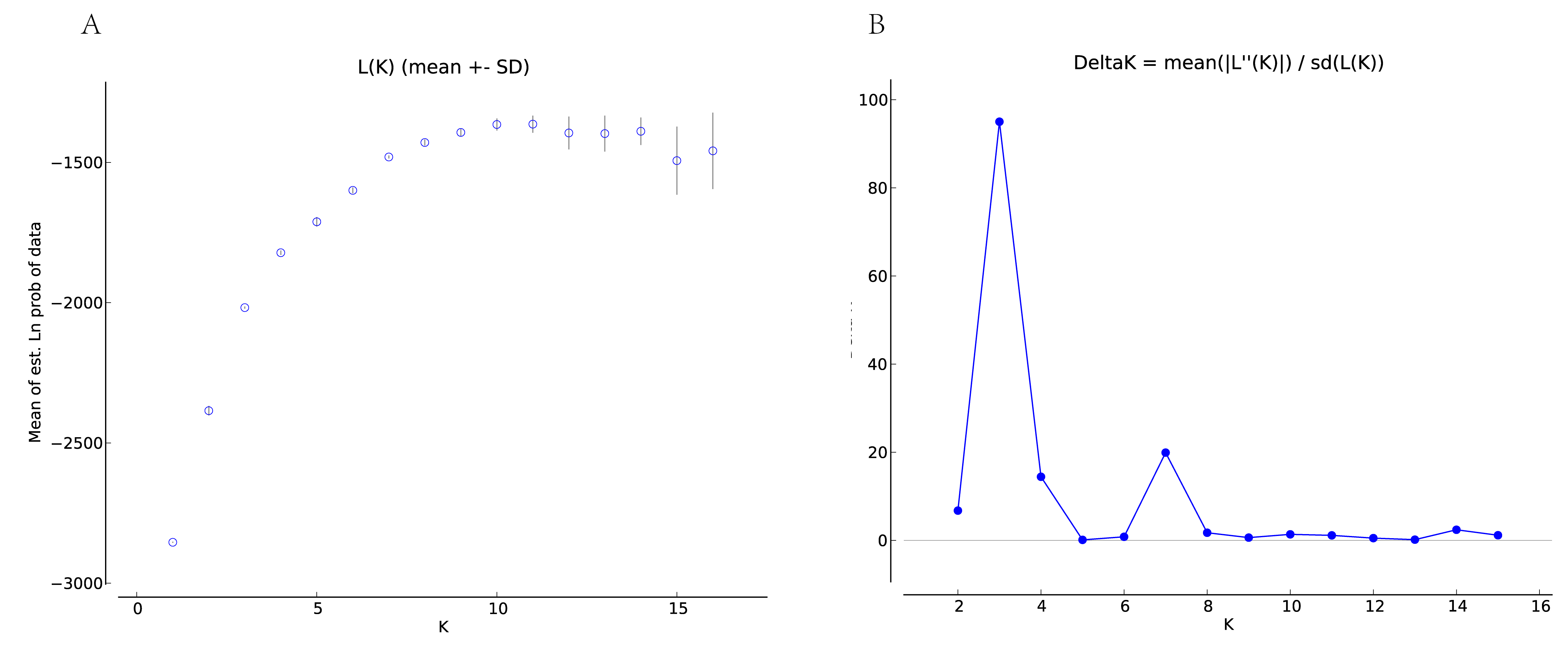

Supplement: FIGURE S2 — Bayesian inference analysis of nuclear data to determine the most likely number of clusters (K) for the four pine species. Distributions of the likelihood L (K) values (A) and delta K values (B) are presented for K = 1–16. [file Image_2.TIF]

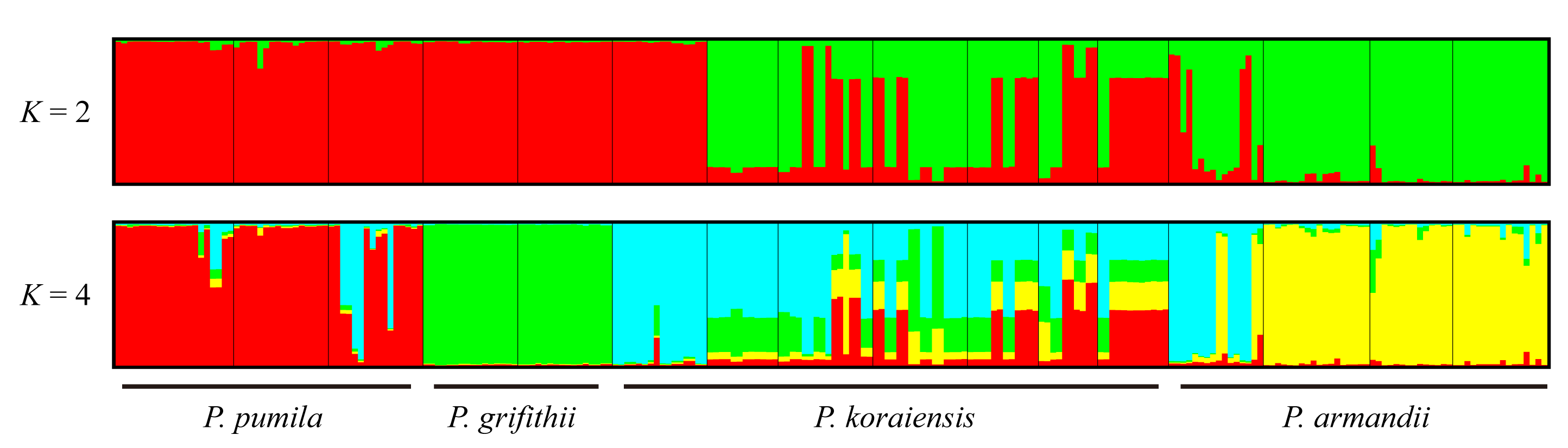

Supplement: FIGURE S3 — Probability of assignments of four closely related pine species into two and four ancestral clusters (K = 2 and K = 4) estimated by the STRUCTURE program. [file Image_3.TIF]

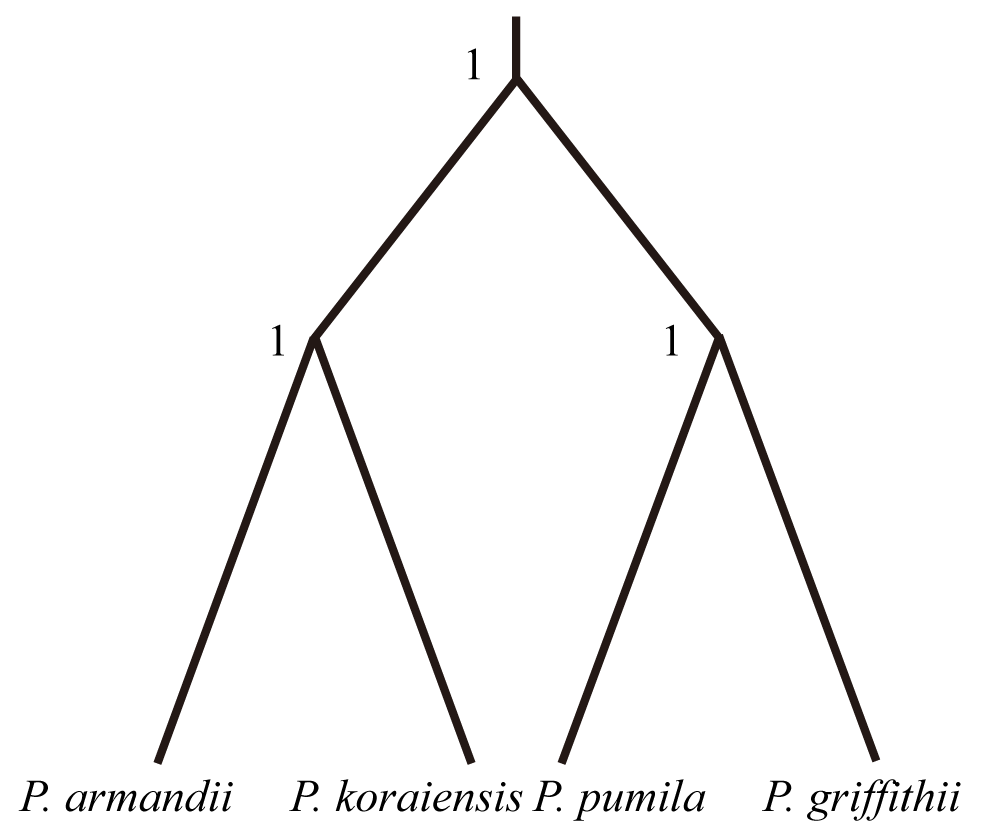

Supplement: FIGURE S4 — Dendrogram derived for four closely related pines species using BPP with six nuclear loci sequences. Bootstrap values are shown above each branch in the BPP tree. [file Image_4.TIF]

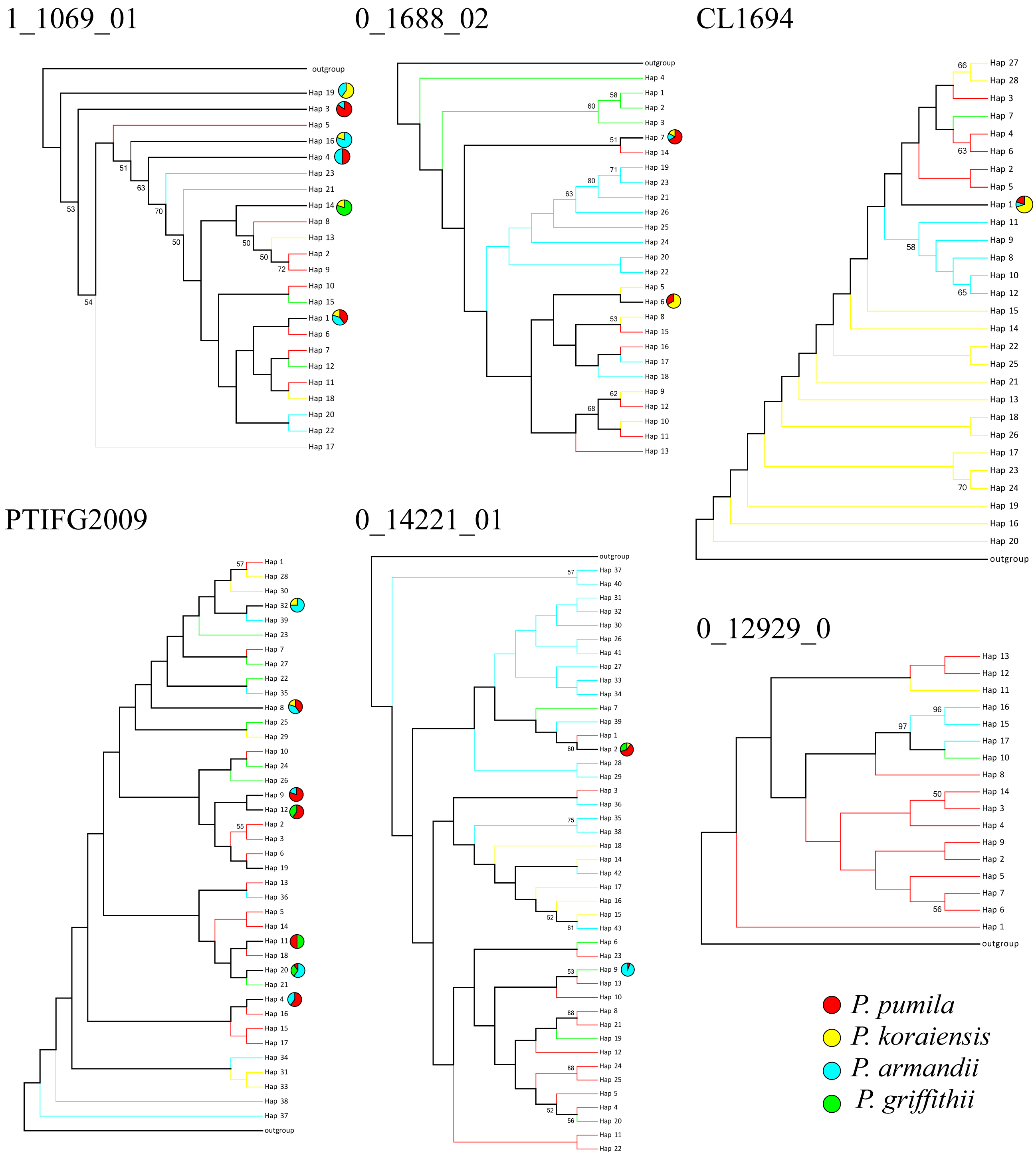

Supplement: FIGURE S5 — Maximum likelihood (ML) tree based on the nuclear haplotypes built using PAUP version 4.0b10. Pinus bungeana was designated as an outgroup. Bootstrap values for ML analyses are shown above branch in the trees. Pie charts indicate the probabilities of the haplotypes for each species. [file Image_5.TIF]
