# Supplementary material for: Effects of Geological and Environmental Events on the Diversity and Genetic Divergence of Four Closely Related Pines: Pinus koraiensis, P. armandii, P. griffithii, and P. pumila
Source: Front Plant Sci. 2018 Aug 28;9:1264. doi: 10.3389/fpls.2018.01264 (PMC6121107; doi:10.3389/fpls.2018.01264)
Supplement: TABLE S1 — Sampling locality and altitude for Pinus pumila, P. griffithii, P. koraiensis, and P. armandii. [file Table_1.DOC]

**Table S1** Sampling locality and altitude for *Pinus pumila*, *P. griffithii*, *P. koraiensis*, and *P. armandii*.

| Species | Population | Location | Latitude | Longitude | Altitude (m) | n |
| --- | --- | --- | --- | --- | --- | --- |
| *P. armandii* | BM | Baotianman HN | 33.57 | 111.98 | 727 | 14 |
|  | MM | Mianming SC | 28.51 | 102.21 | 1932 | 12 |
|  | JO | Jiulong SC | 29.41 | 101.94 | 2918 | 12 |
|  | AK | Ankang SX | 32.23 | 108.87 | 1980 | 10 |
| *P. pumila* | MH | Mohe HL | 52.37 | 122.47 | 862 | 16 |
|  | GH | Genhe NM | 51.84 | 122.04 | 847 | 16 |
|  | HL | Greater Hinggan HL | 51.62 | 124.00 | 627 | 16 |
| *P. koraiensis* | YC | Yinchun HL | 47.20 | 128.95 | 386 | 16 |
|  | FS | Fusong JL | 42.56 | 127.78 | 769 | 12 |
|  | TL | Tieli HL | 46.95 | 128.88 | 418 | 10 |
|  | DH | Dunhua JL | 48.51 | 128.59 | 594 | 10 |
|  | NG | Ningan HL | 44.20 | 126.53 | 900 | 12 |
|  | BS | Baishan JL | 41.94 | 127.59 | 829 | 16 |
|  | DD | Dandong LN | 40.13 | 124.37 | 40 | 14 |
| *P. griffithii* | YD | Yadong XZ | 27.43 | 88.90 | 3390 | 16 |
|  | JI | Jilong XZ | 28.51 | 85.22 | 3362 | 14 |

n: number of individuals

HN: Henan

SC: Sichuan

SX: Shaanxi

NM: Inner Mongolia

HL: Heilongjiang

JL: Jilin

LN: Liaoning

XZ: Tibet
