# Supplementary material for: Effects of Geological and Environmental Events on the Diversity and Genetic Divergence of Four Closely Related Pines: Pinus koraiensis, P. armandii, P. griffithii, and P. pumila
Source: Front Plant Sci. 2018 Aug 28;9:1264. doi: 10.3389/fpls.2018.01264 (PMC6121107; doi:10.3389/fpls.2018.01264)
Supplement: TABLE S2 — Details of the primers used in this study. [file Table_2.DOC]

**Table S2** Details of the primers used in this study.

| No. | Locus | Primer sequence(5’-3’) | Length (bp) | Tm (ºC) | Putative function | Reference |
| --- | --- | --- | --- | --- | --- | --- |
| 1 | 1_1609_01 | F:CCTCAATGATTTACCACC  R:GCAGCTTGTATGTCAG | 387 | 58 | Hypothetical protein | Eckert et al., 2013 |
| 2 | CL1694 | F:AGATGCTGAACAGGTGCAGG  R:TATATACAGTGCTGTCTCGTAGAAGAGG | 328 | 60 | Maternal effect embryo arrest 5 (translation  elongation factor) | Eckert et al., 2013 |
| 3 | PTIFG2009 | F:CACAGTTCCCCACAGCAAC  R:ACAAGCGGTTCAGTGGCTC | 505 | 53 | 60S ribosomal protein L10A | Ma et al., 2006 |
| 4 | 0_12929_02 | F:GTTACAACATCAGCAATCAG  R:AAGCCAATCCACCAGTTATATACAG | 735 | 53 | Protein kinase family protein | Eckert et al., 2013 |
| 5 | 0_14221_01 | F:GATCATTCTAGGCACAGCACAAG  R:CCTGTACCGTGTTCATCAATTTAGCAAG | 539 | 53.5 | Serine-tRNA ligase | Eckert et al., 2013 |
| 6 | 0_1688_02 | F:GACTGACTATAACAGCC  R:CCCAAAATCACCAACAAC | 613 | 58 | Leucine-rich repeat family protein | Eckert et al., 2013 |
