# Supplementary material for: Effects of Geological and Environmental Events on the Diversity and Genetic Divergence of Four Closely Related Pines: Pinus koraiensis, P. armandii, P. griffithii, and P. pumila
Source: Front Plant Sci. 2018 Aug 28;9:1264. doi: 10.3389/fpls.2018.01264 (PMC6121107; doi:10.3389/fpls.2018.01264)
Supplement: TABLE S3 — Distribution of polymorphic sites in the four related pine species: Pinus pumila, P. griffithii, P. koraiensis, and P. armandii. S1, number of exclusive polymorphic sites in the first species; S2, number of exclusive polymorphic sites in the second species; SS, number of shared polymorphisms; Sf, number of fixed differences between two species. [file Table_3.DOC]

**Table S3** Distribution of polymorphic sites in the four related pine species: *Pinus pumila*, *P. griffithii,* *P. koraiensis*, and *P. armandii*. *S*_1_, number of exclusive polymorphic sites in the first species; *S*_2_, number of exclusive polymorphic sites in the second species; *S*_S_, number of shared polymorphisms; *S*_f_, number of fixed differences between two species.

| Group | *P. pumila* vs *P. griffithii* | | | | *P. pumila* vs *P. koraiensis* | | | | | *P. pumila* vs *P. armandii* | | | | | *P. griffithii* vs *P. koraiensis* | | | | | *P. griffithii* vs *P. armandii* | | | | | *P. koraiensis* vs *P. armandii* | | | | |
| --- | --- | --- | --- | --- | --- | --- | --- | --- | --- | --- | --- | --- | --- | --- | --- | --- | --- | --- | --- | --- | --- | --- | --- | --- | --- | --- | --- | --- | --- |
|  | *S*_1_ | *S*_2_ | *S*_S_ | *S*_f_ | | *S*_1_ | *S*_2_ | *S*_S_ | *S*_f_ | | *S*_1_ | *S*_2_ | *S*_S_ | *S*_f_ | | *S*_1_ | *S*_2_ | *S*_S_ | *S*_f_ | | *S*_1_ | *S*_2_ | *S*_S_ | *S*_f_ | | *S*_1_ | *S*_2_ | *S*_S_ | *S*_f_ |
| 1_1609_01 | 13 | 2 | 1 | 0 | | 8 | 1 | 6 | 0 | | 7 | 2 | 7 | 0 | | 2 | 6 | 1 | 0 | | 2 | 8 | 1 | 0 | | 1 | 3 | 6 | 0 |
| 0_1688_02 | 6 | 10 | 0 | 1 | | 4 | 3 | 2 | 0 | | 5 | 9 | 1 | 0 | | 9 | 4 | 1 | 0 | | 10 | 10 | 0 | 0 | | 3 | 8 | 2 | 0 |
| PTIFG2009 | 6 | 2 | 7 | 0 | | 5 | 3 | 8 | 0 | | 3 | 2 | 10 | 0 | | 6 | 8 | 3 | 0 | | 3 | 6 | 6 | 0 | | 3 | 4 | 8 | 0 |
| 0_12929_02 | 14 | 0 | 0 | 4 | | 14 | 0 | 0 | 0 | | 14 | 4 | 0 | 4 | | 0 | 0 | 0 | 5 | | 0 | 4 | 0 | 0 | | 0 | 4 | 0 | 5 |
| 0_14221_01 | 18 | 3 | 1 | 0 | | 18 | 5 | 3 | 0 | | 18 | 13 | 3 | 0 | | 3 | 6 | 0 | 0 | | 3 | 14 | 0 | 0 | | 6 | 13 | 2 | 0 |
| CL1694 | 5 | 0 | 0 | 1 | | 5 | 20 | 0 | 0 | | 5 | 6 | 0 | 0 | | 0 | 20 | 0 | 1 | | 0 | 6 | 0 | 1 | | 18 | 4 | 2 | 0 |
| average | 10 | 3 | 2 | 1 | | 9 | 5 | 3 | 0 | | 9 | 6 | 4 | 1 | | 3 | 7 | 1 | 1 | | 3 | 8 | 1 | 0 | | 5 | 6 | 3 | 1 |
