# Supplementary material for: Effects of Geological and Environmental Events on the Diversity and Genetic Divergence of Four Closely Related Pines: Pinus koraiensis, P. armandii, P. griffithii, and P. pumila
Source: Front Plant Sci. 2018 Aug 28;9:1264. doi: 10.3389/fpls.2018.01264 (PMC6121107; doi:10.3389/fpls.2018.01264)
Supplement: TABLE S4 — Maximum frequency of derived mutations (MFDM) test results for the four pine species. [file Table_4.DOC]

| **Table S4** Maximum frequency of derived mutations (MFDM) test results for the four pine species. | | | | |
| --- | --- | --- | --- | --- |
| Locus | *P*-value | | | |
|  | *P. pumila* | *P. griffithii* | *P. koraiensis* | *P. armandii* |
| 1_1609_01 | 0.358974 (0.052) | 0.444444 (0.075) | 0.545455 (0.182) | 0.102564 (0.052) |
| CL1694 | 1.0 (0.075) | - | 0.611765 (0.05) | 0.470588 (0.118) |
| PtIFG2009 | 0.04674^*^ (0.00625^**^) | 0.08 (0.016^*^) | 0.08 (0.04^*^) | 0.08 (0.016^*^) |
| 0_1688_02 | 0.358974 (0.052) | 0.24 (0.08) | 1.0 (0.223) | 0.12766 (0.05) |
| 0_12929_02 | 1.0 (0.05) | - | - | 1.0 (0.08) |
| 0_14221_01 | 0.848485 (0.061) | 0.62069 (0.07) | 0.181818 (0.0455^*^) | 0.075472 (0.05) |

Note: the values outside the brackets represent *P*-values (Significant level: **P* < 0.05, ***P* < 0.01). The values in brackets represent the *P*-values in existence of recombination events.
