# Supplementary material for: Effects of Geological and Environmental Events on the Diversity and Genetic Divergence of Four Closely Related Pines: Pinus koraiensis, P. armandii, P. griffithii, and P. pumila
Source: Front Plant Sci. 2018 Aug 28;9:1264. doi: 10.3389/fpls.2018.01264 (PMC6121107; doi:10.3389/fpls.2018.01264)
Supplement: TABLE S5 — Analysis of molecular variance (AMOVA) for nucleotide sequence variations in four Pinus species. [file Table_5.DOC]

**Table S5** Analysis of molecular variance (AMOVA) for nucleotide sequence variations in four *Pinus* species.

| Locus | Source of variation | d. f. | SS | VC | Variation ( % ) | Fixation index |
| --- | --- | --- | --- | --- | --- | --- |
| 1_1609_01 | Among groups | 3 | 9.524 | 0.050 | 4.18 | *F*_CT_ = 0.042 |
|  | Among populations within groups | 6 | 10.157 | 0.052 | 4.37 | *F*_ST_ = 0.085^***^ |
|  | Within populations | 110 | 120.036 | 1.091 | 91.45 | *F*_SC_ = 0.046 |
|  | Total | 119 | 139.717 | 1.193 |  |  |
| 0_1688_02 | Among groups | 3 | 98.524 | 0.910 | 35.66 | *F*_CT_ = 0.357^***^ |
|  | Among populations within groups | 7 | 44.435 | 0.473 | 18.52 | *F*_ST_ = 0.542^***^ |
|  | Within populations | 113 | 132.12 | 1.169 | 45.82 | *F*_SC_ = 0.289^***^ |
|  | Total | 123 | 275.097 | 2.552 |  |  |
| CL1694 | Among groups | 3 | 54.970 | 0.497 | 26.14 | *F*_CT_ = 0.261^***^ |
|  | Among populations within groups | 8 | 42.761 | 0.371 | 19.47 | *F*_ST_ = 0.456^***^ |
|  | Within populations | 128 | 132.470 | 1.035 | 54.39 | *F*_SC_ = 0.264^***^ |
|  | Total | 139 | 230.200 | 1.903 |  |  |
| PTIFG2009 | Among groups | 3 | 118.099 | 1.065 | 37.79 | *F*_CT_ = 0.398^***^ |
|  | Among populations within groups | 5 | 41.203 | 0.527 | 18.70 | *F*_ST_ =0.565^***^ |
|  | Within populations | 115 | 136.023 | 1.225 | 43.51 | *F*_SC_ =0.301^***^ |
|  | Total | 123 | 295.325 | 2.863 |  |  |
| 0_12929_02 | Among groups | 3 | 128.410 | 1.775 | 71.82 | *F*_CT_ = 0.718^***^ |
|  | Among populations within groups | 5 | 2.994 | –0.009 | –0.38 | *F*_ST_ = 0.714^***^ |
|  | Within populations | 97 | 68.483 | 0.706 | 28.56 | *F*_SC_ = –0.013^***^ |
|  | Total | 105 | 199.887 | 2.472 |  |  |
| 0_14221_01 | Among groups | 3 | 134.750 | 1.335 | 45.64 | *F*_CT_ = 0.456^***^ |
|  | Among populations within groups | 6 | 27.368 | 0.249 | 8.50 | *F*_ST_ = 0.541^***^ |
|  | Within populations | 120 | 160.967 | 1.341 | 45.86 | *F*_SC_ = 0.156^***^ |
|  | Total | 129 | 323.085 | 2.925 |  |  |
| All locus | Among groups | 3 | 71157.13 | 347.039 | 38.88 | *F*_CT_ = 0.389^***^ |
|  | Among populations within groups | 12 | 41453.963 | 209.651 | 23.49 | *F*_ST_ = 0.624^***^ |
|  | Within populations | 226 | 75896.613 | 335.826 | 37.63 | *F*_SC_ = 0.383^***^ |
|  | Total |  | 188507.707 | 892.515 |  |  |

d.f., degrees of freedom; SS, sum of squares; VC, variance components

Significance levels: **P* < 0.05, ** *P* < 0.01, and ****P* < 0.001; 1000 permutations

*F*_CT_, divergence among groups within four species

*F*_ST_, divergence among populations within four species

*F*_SC_, divergence among individuals within populations of four species
