# Supplementary material for: Effects of Geological and Environmental Events on the Diversity and Genetic Divergence of Four Closely Related Pines: Pinus koraiensis, P. armandii, P. griffithii, and P. pumila
Source: Front Plant Sci. 2018 Aug 28;9:1264. doi: 10.3389/fpls.2018.01264 (PMC6121107; doi:10.3389/fpls.2018.01264)
Supplement: TABLE S6 — Genetic divergence (FST) at each locus among specie based on pairwise comparisons for P. armandii, P. griffithii, P. koraiensis and P. pumila. [file Table_6.DOC]

**Table S6** Genetic divergence (*F*_ST_) at each locus among specie based on pairwise comparisons for *P. armandii*, *P. griffithii*, *P. koraiensis* and *P. pumila*.

| Group | *P. pumila* vs *P. griffithii* | *P. pumila* vs *P. koraiensis* | *P. pumila* vs *P. armandii* | *P. griffithii* vs *P. koraiensis* | *P. griffithii* vs *P. armandii* | *P. koraiensis* vs *P. armandii* |
| --- | --- | --- | --- | --- | --- | --- |
| 1_1609_01 | 0.341 | 0.008 | 0.033 | 0.380 | 0.400 | 0.028 |
| 0_1688_02 | 0.622 | 0.406 | 0.369 | 0.424 | 0.427 | 0.283 |
| PTIFG2009 | 0.241 | 0.550 | 0.119 | 0.674 | 0.372 | 0.609 |
| 0_12929_02 | 0.815 | 0.536 | 0.726 | 1.000 | 0.348 | 0.875 |
| 0_14221_01 | 0.298 | 0.446 | 0.234 | 0.747 | 0.660 | 0.632 |
| CL1694 | 0.120 | 0.327 | 0.362 | 0.369 | 0.457 | 0.398 |
