# Supplementary material for: Effects of Geological and Environmental Events on the Diversity and Genetic Divergence of Four Closely Related Pines: Pinus koraiensis, P. armandii, P. griffithii, and P. pumila
Source: Front Plant Sci. 2018 Aug 28;9:1264. doi: 10.3389/fpls.2018.01264 (PMC6121107; doi:10.3389/fpls.2018.01264)
Supplement: TABLE S7 — ΦST values over all loci among species. [file Table_7.DOC]

**Table S7** *Φ*_ST_ values over all loci among species.

|  | *P. pumila* | *P. griffithii* | *P. koraiensis* | *P. armandii* |
| --- | --- | --- | --- | --- |
| *P. pumila* | - |  |  |  |
| *P. griffithii* | 0.18941^***^ | - |  |  |
| *P. koraiensis* | 0.57118^***^ | 0.62006 ^***^ | - |  |
| *P. armandii* | 0.41810 ^***^ | 0.42495 ^***^ | 0.47436^***^ | - |

Significance levels: ***P* < 0.01; ****P* < 0.001
