# Supplementary material for: Effects of Geological and Environmental Events on the Diversity and Genetic Divergence of Four Closely Related Pines: Pinus koraiensis, P. armandii, P. griffithii, and P. pumila
Source: Front Plant Sci. 2018 Aug 28;9:1264. doi: 10.3389/fpls.2018.01264 (PMC6121107; doi:10.3389/fpls.2018.01264)
Supplement: TABLE S8 — Nucleotide variations in Pinus pumila, P. griffithii, P. koraiensis, and P. armandii with equal sample sizes. [file Table_8.DOC]

**Table S8** Nucleotide variations in *Pinus pumila*, *P. griffithii*, *P. koraiensis*, and *P. armandii* with equal sample sizes.

| Species | Locus | Total | | | | | Nonsynonymous Sites | | | | Silent Sites | | | | *R*m |
| --- | --- | --- | --- | --- | --- | --- | --- | --- | --- | --- | --- | --- | --- | --- | --- |
|  |  | *N* | L | S(Singl) | *θ* | *π*_t_ | L | S | *θ_W_* | *π*_a_ | L | S | *θ_W_* | *π*_Sil_ |  |
| *P. pumila* | 1_1609_01 | 56 | 387 | 3 (0) | 0.00204 | 0.00223 | 296 | 3 | 0.00260 | 0.00285 | 79 | 0 | 0.00000 | 0.00000 | 1 |
|  | PTIFG2009 | 56 | 505 | 13 (1) | 0.00662 | 0.00602 | 393 | 10 | 0.00653 | 0.00591 | 111 | 3 | 0.00696 | 0.00646 | 5 |
|  | 0_1688_02 | 56 | 613 | 4 (0) | 0.00170 | 0.00220 | 471 | 2 | 0.00109 | 0.00163 | 132 | 2 | 0.00390 | 0.00425 | 0 |
|  | CL1694 | 56 | 305 | 5 (2) | 0.00421 | 0.00330 | 59 | 0 | 0.00000 | 0.00000 | 245 | 5 | 0.00524 | 0.00410 | 0 |
|  | 0_12929_02 | 56 | 735 | 13 (1) | 0.00455 | 0.00238 | 586 | 7 | 0.00307 | 0.00116 | 149 | 6 | 0.01038 | 0.00718 | 2 |
|  | 0_14221_01 | 56 | 585 | 14 (1) | 0.00665 | 0.00724 | 449 | 13 | 0.00744 | 0.00822 | 124 | 2 | 0.00414 | 0.00409 | 3 |
| Average |  | 56 |  | 8.67 | 0.00430 | 0.00390 | 375.67 | 5.8 | 0.00350 | 0.00330 | 140 | 3 | 0.00510 | 0.00435 | 2 |
| *P. griffithii* | 1_1609_01 | 16 | 387 | 1 (0) | 0.00103 | 0.00066 | 294 | 0 | 0.00000 | 0.00000 | 78 | 1 | 0.00496 | 0.00321 | 0 |
|  | PTIFG2009 | 16 | 505 | 5 (2) | 0.00382 | 0.00375 | 393 | 5 | 0.00490 | 0.00481 | 111 | 0 | 0.00000 | 0.00000 | 0 |
|  | 0_1688_02 | 16 | 613 | 1 (0) | 0.00064 | 0.00041 | 471 | 1 | 0.00082 | 0.00053 | 132 | 0 | 0.00000 | 0.00000 | 0 |
|  | CL1694 | 16 | 305 | 0 (0) | 0.00000 | 0.00000 | 59 | 0 | 0.00000 | 0.00000 | 245 | 0 | 0.00000 | 0.00000 | - |
|  | 0_12929_02 | 16 | 735 | 0 (0) | 0.00000 | 0.00000 | 290 | 0 | 0.00000 | 0.00000 | 441 | 0 | 0.00000 | 0.00000 | - |
|  | 0_14221_01 | 16 | 585 | 1 (0) | 0.00066 | 0.00043 | 455 | 1 | 0.00085 | 0.00055 | 127 | 0 | 0.00000 | 0.00000 | 0 |
| Average |  | 16 |  | 1.33 | 0.00103 | 0.00088 | 327 | 1.2 | 0.00110 | 0.00098 | 189 | 0.17 | 0.00083 | 0.00054 | 0 |
| *P. koraiensis* | 1_1609_01 | 20 | 387 | 6 (0) | 0.00548 | 0.00540 | 303 | 4 | 0.00466 | 0.00506 | 81 | 2 | 0.00874 | 0.00689 | 0 |
|  | PTIFG2009 | 20 | 505 | 4 (2) | 0.00280 | 0.00304 | 393 | 3 | 0.00270 | 0.00271 | 111 | 1 | 0.00320 | 0.00422 | 0 |
|  | 0_1688_02 | 20 | 613 | 5 (0) | 0.00292 | 0.00374 | 217 | 0 | 0.00000 | 0.00000 | 387 | 5 | 0.00456 | 0.00585 | 1 |
|  | CL1694 | 20 | 305 | 0 (0) | 0.00000 | 0.00000 | 228 | 0 | 0.00000 | 0.00000 | 75 | 0 | 0.00000 | 0.00000 | - |
|  | 0_12929_02 | 20 | 735 | 0 (0) | 0.00000 | 0.00000 | 287 | 0 | 0.00000 | 0.00000 | 444 | 0 | 0.00000 | 0.00000 | - |
|  | 0_14221_01 | 20 | 539 | 6 (3) | 0.00425 | 0.00317 | 453 | 5 | 0.00390 | 0.00285 | 123 | 2 | 0.00575 | 0.00452 | 1 |
| Average |  | 20 |  | 3.5 | 0.00258 | 0.00256 | 313.5 | 2 | 0.00188 | 0.00177 | 203.5 | 1.67 | 0.00371 | 0.00358 | 1 |
| *P. armandii* | 1_1609_01 | 36 | 387 | 5 (1) | 0.00376 | 0.00326 | 306 | 4 | 0.00384 | 0.00347 | 81 | 1 | 0.00359 | 0.00259 | 0 |
|  | PTIFG2009 | 36 | 505 | 11 (4) | 0.00656 | 0.00493 | 393 | 9 | 0.00545 | 0.00527 | 111 | 2 | 0.00689 | 0.00485 | 2 |
|  | 0_1688_02 | 36 | 613 | 9 (0) | 0.00432 | 0.00497 | 471 | 6 | 0.00371 | 0.00385 | 133 | 3 | 0.00658 | 0.00908 | 2 |
|  | CL1694 | 36 | 305 | 6 (0） | 0.00572 | 0.00514 | 59 | 1 | 0.00494 | 0.00355 | 245 | 5 | 0.00593 | 0.00555 | 0 |
|  | 0_12929_02 | 36 | 735 | 4 (1) | 0.00158 | 0.00229 | 586 | 4 | 0.00198 | 0.00287 | 146 | 0 | 0.00000 | 0.00000 | 0 |
|  | 0_14221_01 | 36 | 539 | 7 (1) | 0.00348 | 0.00328 | 456 | 5 | 0.00319 | 0.00295 | 126 | 2 | 0.00462 | 0.00457 | 3 |
| Average |  | 36 |  | 7 | 0.00424 | 0.00398 | 378.5 | 4.8 | 0.00385 | 0.00366 | 140.3 | 2.17 | 0.00460 | 0.00444 | 1 |

*N*, number of individuals; *L*, length in base pairs; *S*, number of segregating sites; Singl, number of singleton mutations; *θ_W_*, Watterson’s parameter (Watterson 1975); *π*_t_, nucleotide diversity across all loci; *π*_a_, nucleotide diversity at nonsynonymous sites; *π*_sil_, nucleotide diversity at silent sites; *R*m, minimum number of recombinant events.
